# Molecular Dynamics Simulation of the Allosteric Regulation of eIF4A Protein from the Open to Closed State, Induced by ATP and RNA Substrates

**DOI:** 10.1371/journal.pone.0086104

**Published:** 2014-01-23

**Authors:** Hongqing Meng, Chaoqun Li, Yan Wang, Guangju Chen

**Affiliations:** Key Laboratory of Theoretical and Computational Photochemistry, Ministry of Education, College of Chemistry, Beijing Normal University, Beijing, China; Instituto de Tecnologica Química e Biológica, UNL, Portugal

## Abstract

**Background:**

Eukaryotic initiation factor 4A (eIF4A) plays a key role in the process of protein translation initiation by facilitating the melting of the 5′ proximal secondary structure of eukaryotic mRNA for ribosomal subunit attachment. It was experimentally postulated that the closed conformation of the eIF4A protein bound by the ATP and RNA substrates is coupled to RNA duplex unwinding to promote protein translation initiation, rather than an open conformation in the absence of ATP and RNA substrates. However, the allosteric process of eIF4A from the open to closed state induced by the ATP and RNA substrates are not yet fully understood.

**Methodology:**

In the present work, we constructed a series of diplex and ternary models of the eIF4A protein bound by the ATP and RNA substrates to carry out molecular dynamics simulations, free energy calculations and conformation analysis and explore the allosteric properties of eIF4A.

**Results:**

The results showed that the eIF4A protein completes the conformational transition from the open to closed state via two allosteric processes of ATP binding followed by RNA and vice versa. Based on cooperative allosteric network analysis, the ATP binding to the eIF4A protein mainly caused the relative rotation of two domains, while the RNA binding caused the proximity of two domains via the migration of RNA bases in the presence of ATP. The cooperative binding of ATP and RNA for the eIF4A protein plays a key role in the allosteric transition.

## Introduction

Initiation of translation in eukaryotes is regarded as rate-limiting for protein synthesis. It requires eukaryotic translation initiation factors (eIFs) for recruiting ribosomal subunits to messenger ribonucleic acids (mRNAs) [1]. Eukaryotic translation initiation factor 4F (eIF4F) plays a key role in translation initiation, and includes three subunits, i.e., the mRNA cap-binding protein eIF4E, large scaffolding protein eIF4G and RNA helicase eIF4A. The eIF4F complex functions to unwind local secondary structures of mRNA, to create a ribosome landing pad [2]–[8]. The RNA helicase eIF4A, i.e., eukaryotic initiation factor 4A, is a member of DEA (D/H)-box RNA helicase family of proteins. Helicases interact with RNA during RNA splicing, ribosome biogenesis, and RNA degradation [9]–[11]. The eIF4A protein, as an RNA-dependent ATPase and ATP-dependent RNA helicase, is thought to facilitate ribosomal attachment in the cytoplasm by melting the 5′ proximal secondary structure of eukaryotic mRNAs [12]–[14]. Specific DEA (D/H)-box proteins use the energy from RNA-dependent adenosine triphosphate (ATP) hydrolysis to drive the endothermic strand unwinding of mRNAs [15], [16]. It was postulated that the closed conformation of the eIF4A protein, in the presence of ATP and RNA substrates, is coupled to RNA distortion and double-strand destabilization [17]–[19]. Therefore, studies pertaining to the structural characteristics and the eIF4A activity in the protein translation initiation have become a hot topic in recent years [17]–[23].

The eIF4A is the prototypic member of the DEAD-box protein family and represents the minimal helicase core. The first X-ray structure of amino-terminal domain (assigned as N-domain including the residues 1–222) of yeast eIF4A protein was determined respectively by Benz et al. (PDB: 1QVA) and Johnson et al. (PDB: 1QDE) in 1999 [24], [25]. The carboxyl-terminal domain (assigned as C-domain including the residues 233–394) and full-length eIF4A protein in open state have been reported by Caruthers et al. (PDB: 1FUK, 1FUU) in 2000 [26]. Each of two domains in the eIF4A protein consists of an nα-mβ-lα fold structure. The n/l and m, respectively, represent the numbers of α helices and β strands. The mβ sheet is located at the center flanked by nα helices on one side and lα helices on the other side. The N-domain and C-domain are formed respectively by the 5α-8β-4α fold in the order α(1-5)-β(1-8-2-7-6-3-5-4)-α(6-9)) and the 2α-7β-4α fold in the order α(1-2)-β(3-4-2-5-6-1-7)-α(3-6)) [24]–[26]. The two domains in the open eIF4A state are connected by an extended linker of 223–233 residues. The end-to-end length of the eIF4A protein in the open state is about 80 Å, ∼18 Å of which is accounted for by the linker [26]. However, the X-ray structure of closed yeast eIF4A protein bound by ATP and RNA substrates has yet to be determined. Further, the yeast eIF4A protein exhibits 62% homology at the amino acid level with the human eukaryotic translation initiation factor 4AIII (eIF4AIII) [18]. It has been proposed that the closed conformation of all DEAD-box proteins was similar to the structure of closed human eIF4AIII [17], [23]. The X-ray structure of closed human eIF4AIII protein bound by ADPNP and RNA substrates was reported respectively by Andersen et al (PDB: 2HYI) and Bono et al (PDB: 2J0S) in 2006 [17], [18]. The closed human eIF4AIII protein differs structurally from the open conformation of yeast eIF4A protein in that the two domains in closed state are in close proximity to each other and extensively interconnected. They interact with ATP and RNA substrates with high affinity [17]–[19], [27]–[29]. Previous studies indicated that cooperative binding of ATP and RNA to the eIF4A protein triggers the formation of the closed conformation, as a hydrolysis and unwinding-competent state, leading to the assemblies of the catalytic sites and the bipartite RNA binding sites in both domains [19], [30]–[33]. A kink in the backbone of the bound RNA sequence in a closed state locally destabilizes the duplex to dissociate an RNA strand [34], [35]. Presumably, the eIF4A protein resumes an open state upon ATP dephosphorylation, resetting it for another catalytic cycle [21], [36], [37]. These studies suggest that eIF4A acts as a nucleotide-dependent switch that alternates between open and closed conformations during the catalytic cycle of duplex separation [21], [30], [38]. Therefore, the process of the eIF4A protein change from an open conformation to a closed one is very important to the RNA unwinding catalytic cycle. Herschlag and Klostermeier and their co-workers proposed that the eIF4A enzyme binds ATP and RNA substrates randomly [30], [32]. Furthermore, no experimental structures of both the open and the closed conformations have been determined for either the yeast or the human eIF4A protein [26], [32]. The structures of the closed human eIF4AIII and yeast eIF4A are similar. However, the eIF4AIII, which constitutes the RNA-binding platform anchoring other EJC (exon junction complex) components to the spliced mRNA in the nucleus, is quite different functionally from the eIF4A [39]. Therefore, the factors affecting the conformational transition from the open to the closed state for the two eIF4AIII and eIF4A proteins might be different. On the other hand, the allosteric properties of eIF4A protein have been theoretically investigated little until now. The dynamic transition mechanism of eIF4A protein from the open to closed state, mediated by ATP and RNA substrates has yet to be fully understood at the atomic level.

Since the eIF4A protein was a typical member of the DEAD-box protein family [26], we selected the yeast eIF4A to explore the transition mechanisms, to obtain valuable insight into the other RNA helicases. Molecular dynamics simulations and free energy calculations for a series of diplex and ternary models of the eIF4A protein bound by the ATP and RNA substrates were carried out to investigate the binding of ATP followed by RNA and vice versa. These studies may be helpful to obtain valuable insights into the conformational transition mechanism and cooperative binding networks induced by ATP and RNA substrates.

## Models and Methods

### Initial structures

**Figure 1:**
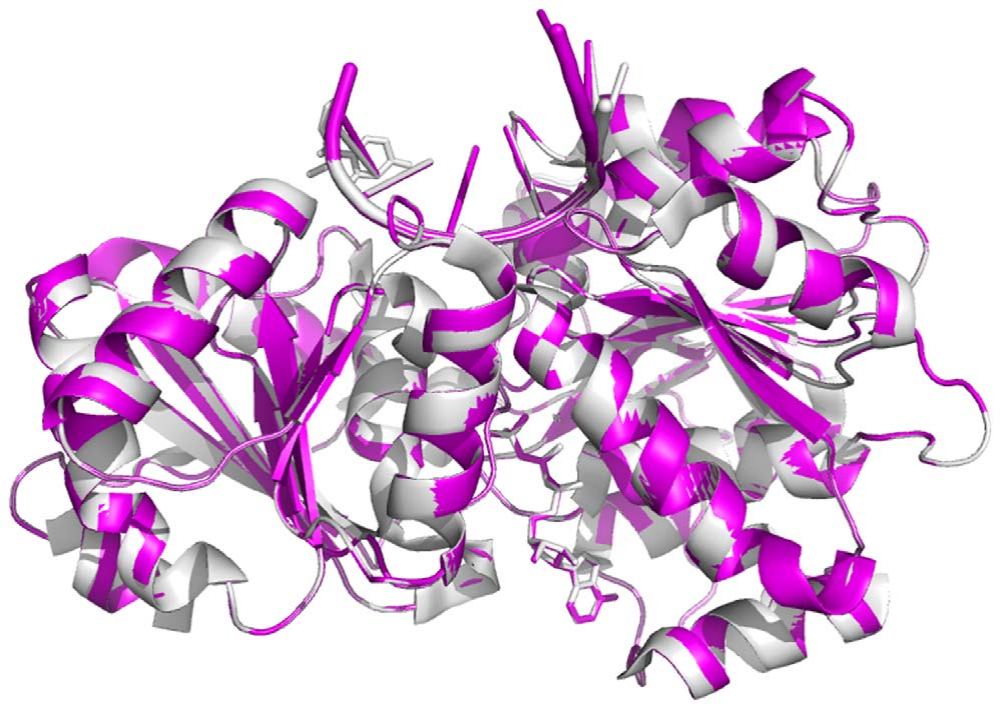
Superposition of two complexes. Superposition of the yeast ATP+RNA+C-eIF4A model (magenta) and the closed human eIF4AIII+ADPNP+RNA complex (gray, PDB-ID 2J0S) in the homology modeling.

Based on the previous experimental studies, the initial structure of the open eIF4A protein (assigned as O-eIF4A model) used in the MD simulations was employed from the X-ray crystal structure (PDB entry 1FUU (B chain)). The O-eIF4A model contains a N-terminal domain of 1–222 residues consisting of 9α helices and 8β strands, a C-terminal domain of 234–394 residues consisting of 6α helices and 7β strands and a linker of two domains consisting of 223–233 residues [26]. The missing residues (i.e. Ser1–Ser10, Arg352–Arg355) in this model were repaired using the loop search method in the Swiss-Pdb Viewer (http://spdbv.vital-it.ch/). Based on the X-ray crystal structure of the human eIF4AIII+ADPNP+RNA complex with a closed conformation (PDB entry 2J0S (A chain)), we build the closed structure of the yeast eIF4A protein bound by ATP and RNA substrates by using the homology modeling technologies in the “Build Mutants protocol” of the Discovery Studio v.2.1 (DS 2.1, 2008) (assigned as ATP+RNA+C-eIF4A model) [40], which is shown in Figure 1. The human eIF4AIII protein shares 62% amino acid sequence identity with the yeast eIF4A protein [18], with highly identical conserved motifs (see Figure S1). Therefore, the structural construction of the closed yeast eIF4A protein by using the closed eIF4AIII template is feasible. In order to investigate the allosteric properties of eIF4A protein bound by ATP substrate or RNA molecule, the ATP+eIF4A or RNA+eIF4A model was built by aligning the crystal structures of open apo eIF4A protein and eIF4AIII+ADPNP+RNA complex using the N- and C- domains superimposed sites. We then imported the coordinates of Mg-ATP or RNA to the open apo eIF4A protein, as a starting structure in the allosteric step O-eIF4A→ATP+eIF4A or O-eIF4A→RNA+eIF4A, respectively. The RNA molecule in the RNA+eIF4A model consists of a 12-uracil single-strand fragment. In order to investigate further allosteric properties of eIF4A second bound by RNA molecule or ATP substrate for the stable ATP+eIF4A or RNA+eIF4A complex, we used the average structure extracted from the equilibrium trajectories of ATP+eIF4A or RNA+eIF4A model, then, respectively combined with RNA or ATP substrate as the initial structure, and used the average structure extracted from the trajectories of ATP+RNA+C-eIF4A model as the target structure to carry out targeted molecular dynamics (TMD) simulations. These two allosteric steps were assigned as the RNA second binding step of (ATP+eIF4A)+RNA→(ATP+RNA+C-eIF4A) and as the ATP second binding step of (RNA+eIF4A)+ATP→(ATP+RNA+C-eIF4A). We selected four intermediate structures of I, II, III, IV from the RNA-second binding TMD simulation or those of I', II', III', IV' from the ATP-second binding based on the average interval of RMSD variation as the starting structures for the CMD simulations in order to obtain the possible equilibrium intermediate structures during the RNA or ATP second binding step. Given that the single RNA strand has some phosphate groups, 43 Na^+^ and 16 Cl^−^ counterions are added to the ATP+RNA+C-eIF4A model to achieve electroneutrality and to satisfy the experimental ionic strength of 100 mM [30]. Similar counterion processes are applied to other models. The systems were explicitly solvated by using the TIP3P water potential inside a rectangular box large enough to ensure the solvent shell extended to 10 Å in all directions of each system studied.

### Molecular dynamics simulation protocols

All CMD and TMD simulations were carried out using the AMBER9 package [41] with a classical AMBER parm99 force field parameters [42], [43] together with the parmbsc0 refinement [44] and gaff force field parameters [45]. Details of CMD and TMD protocols are given in Texts S1 and S2.

### Free-energy analyses

The molecular mechanics Poisson–Boltzmann surface area (MM-PBSA) method [46]–[49] in AMBER9 package was employed to perform the free energy analyses. The binding free energy was computed through calculating the free energy differences of ligand, receptor and their complex as follows:
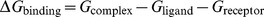
In MM-PBSA, the free energy (*G*) of each state is estimated from molecular mechanical energy *E*_MM_, solvation free energy *G*_SOLV_ and vibrational, rotational, and translational entropies *S*, respectively.
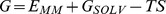


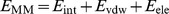


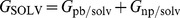
Where T is the temperature; *E*_int_ is internal energy, i.e. the sum of bond, angle, and dihedral energies; *E*_vdw_ is van der Waals energy; *E*_ele_ is electrostatic energy; G_SOLV_ is the sum of electrostatic solvation free energy, *G*_pb/solv_, and the non-polar salvation free energy, *G*_np/solv_. The entropy *S* is estimated by a normal mode analysis of the harmonic vibrational frequencies, calculated using the Nmode module in AMBER9 package [50], [51]. Prior to the normal mode calculations, each structure was fully minimized using a distance dependent dielectric of ε = 4r (r is the distance between two atoms) to mimic the solvent dielectric change from the solute to solvent until the root-mean-square of the elements of the gradient vector was less than 5×10^−4^ kcal·mol^−1^·Å^−1^. Then, the entropy was calculated based on standard statistical mechanics expressions [47], [52]. Computational details are available in Text S3.

### DynDom analyses

DynDom is able to determine dynamic domains, hinge axes, and hinge-bending residues from two protein structures that have different conformations. DynDom generates short segments of the amino-acid chains of these proteins by use of a sliding window and the calculation of the rotation vector associated with the rotation of these segments between the two structures. By treating the components of these rotation vectors as coordinates in a ‘rotation space’, segments that rotate together will have rotation points collocated, indicating possible rigid domains within the structure. Moreover, in creating a domain decomposition, DynDom measures the ratio of interdomain displacement to intradomain displacement as defined by Hayward & Berendsen [53]. For a domain decomposition to be accepted for the hinge axis analysis, this ratio must be larger than 1.0, i.e. there must be more interdomain displacement than intradomain displacement. Thus domains can be identified from the distribution of rotation points. Domains and hinge axes were identified and characterized by using the DYNDOM program (http://www.cmp.uea.ac.uk/dyndom/).

### Calculation of angle between two helices

To analyze conformational changes in the relative orientations of any two helices, the program interhlx (written by Kyoko Yap, available at www.nmr.uhnres.utoronto.ca/ikura/interhlx/) was used to calculate the angle between two helices, including the sign in a structure or a family of structures. The program calculates the sign of the angle between two helices by following this convenient role: The two helices are taken to be positioned by helix I being in front of helix II. Helix I (from N to C) is used to define first vertical vector. A second vertical vector is defined with its tail at the C-terminus of helix II. The angle between helices I and II is the rotation required to align the head of the second vector with the N-terminus of helix II. The vector is rotated in the direction that produces an angle of less than 180 degrees with the clockwise or counterclockwise rotation represented by positive or negative sign. This program can also provide other geometry-based parameters such as interhelical distances [54], [55].

### Fluctuation and correlation analyses

The root-mean-square fluctuations (RMSF) values of residues are a measure of fluctuations and flexibility of backbone Cα of protein over the trajectory broken down by residues in comparison to the average structures [41], [56]. *RMSF_i_* of the Cα atom of each residue was calculated as follows:
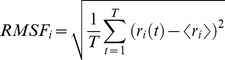
Where *T* is the number of snapshots considered in the time trajectory, *r_i_*(*t*), the position of the Cα atom of residue *i* at time *t*, and 〈*r_i_*〉, the time-averaged position of the Cα atom of residue *i*.

The dynamic feature of a protein and the extent of correlation of the motions of the different regions in a protein were assessed via the calculation of cross-correlation coefficients, *C*(*i,j*) given as follows:
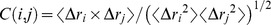
In the equation, Δ*r_i_* and Δ*r_j_* are the displacement vectors for atoms *i* and *j*, respectively, and the angle brackets denotes the ensemble average [41], [56]. In the present study, the correlation coefficients were averaged over the regions of the protein and the resultant cross correlation coefficients are presented in the form of a two-dimensional graph. These structure analyses in the present work were calculated by using PTRAJ module in AMBER9 program [41].

## Results

**Figure 2:**
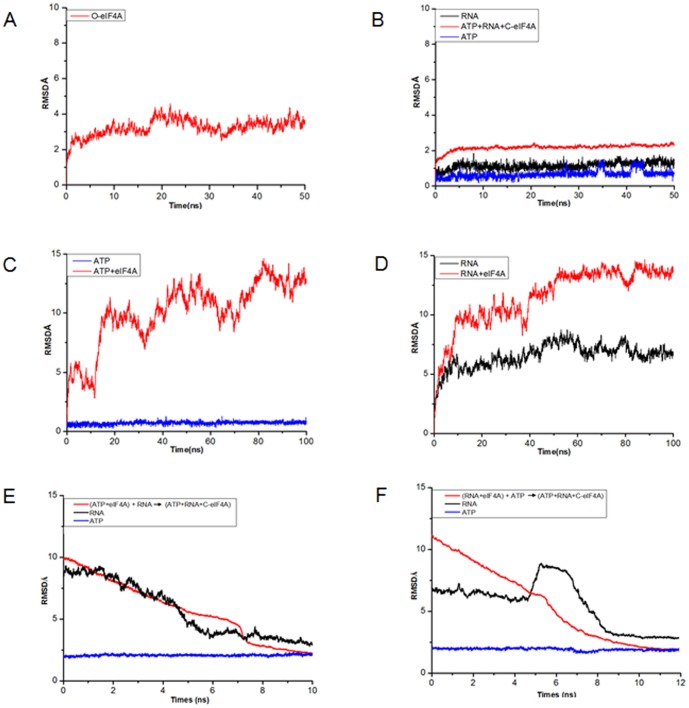
RMSD values of the equilibrium and allosteric models. RMSD values of all backbone atoms with respect to the corresponding starting structures for the CMD simulations of (A) O-eIF4A, (B) ATP+RNA+C-eIF4A, (C) ATP+eIF4A and (D) RNA+eIF4A models, and for the TMD simulations of (E) the (ATP+eIF4A)+RNA→(ATP+RNA+C-eIF4A) transition and (F) the (RNA+eIF4A)+ATP→(ATP+RNA+C-eIF4A) transition.

The root-mean-square deviation (RMSD) values of all the backbone atoms relative to the corresponding starting structures over four trajectories for O-eIF4A, ATP+RNA+C-eIF4A, ATP+eIF4A and RNA+eIF4A models were examined to determine the system equilibrium. It is often considered that small RMSD values of one simulation indicate a stable state of the system and also suggest that the newly constructed models satisfactorily reproduced the experimental structures. However, the large RMSD values suggest large conformational changes of the investigated system. Plots of RMSDs for the two equilibrium systems, O-eIF4A and ATP+RNA+C-eIF4A models, and two allosteric systems, ATP+eIF4A and RNA+eIF4A models, over simulation times are shown in Figure 2A, B, C, D, respectively. As illustrated in Figure 2A and B, the O-eIF4A and ATP+RNA+C-eIF4A systems reached equilibrium after 5 ns, with stable energies during the remainder of each simulation. Therefore, the trajectory analysis of the two systems yielded the equilibrated conformations between 40 ns and 50 ns of simulation time, recording 5000 snapshots at every 2 ps time-interval of each trajectory. It is apparent from Figure 2C and D that the RMSD values of eIF4A protein for the ATP+eIF4A and RNA+eIF4A models presented great variation, which predicts the allosteric properties of eIF4A protein in these two models. Only the first 20 ns (10000 snapshots) of each trajectory for the allosteric ATP+eIF4A and RNA+eIF4A models were used for the structural analysis. Figure 2E and F illustrate the plots for the RMSDs of all backbone atoms in the TMD-simulated structures relative to the final structure of the two allosteric steps of (ATP+eIF4A)+RNA→(ATP+RNA+C-eIF4A) and (RNA+eIF4A)+ATP→(ATP+RNA+C-eIF4A). The backbone atoms in the two TMD simulations reached the same target conformation within 10 ns simulation time.

### 1. Stability of the O-eIF4A and ATP+RNA+C-eIF4A states

**Figure 3:**
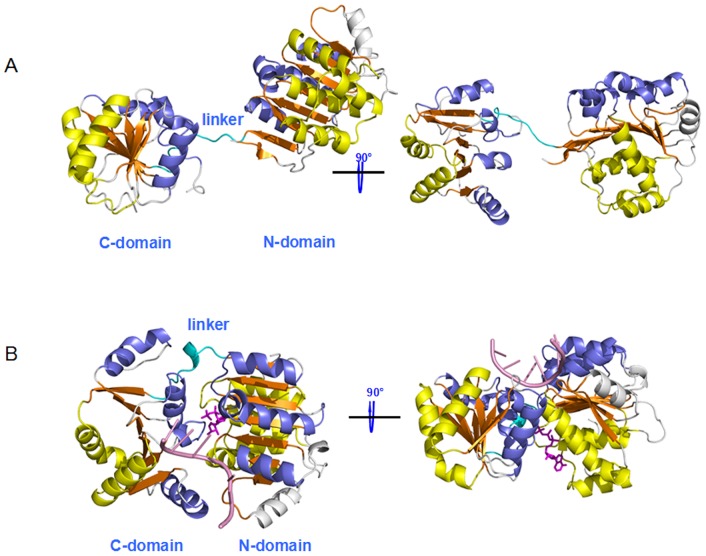
The structures of the open and closed states. MD-simulated overall structures for (A) the open eIF4A state and (B) the closed eIF4A state bound by ATP (colored in magenta) and RNA (colored in pink), the linker in which is colored in cyan.

**Figure 4:**
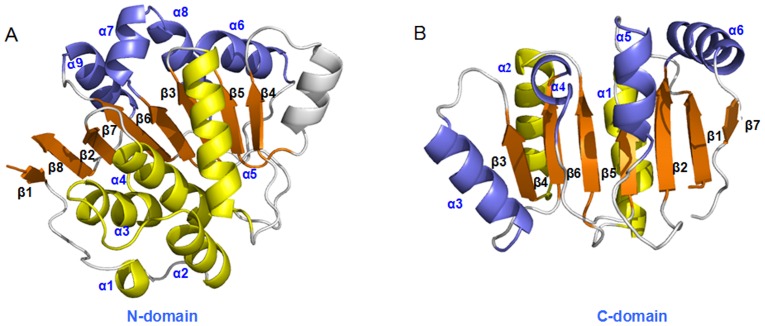
The structures of N- and C- domains of eIF4A. The structures of (A) the N-domain 5α (yellow) - 8β (orange) - 4α (slate) and (B) the C-domain 2α (yellow) - 7β (orange) - 4α (slate) for the eIF4A protein.

**Figure 5:**
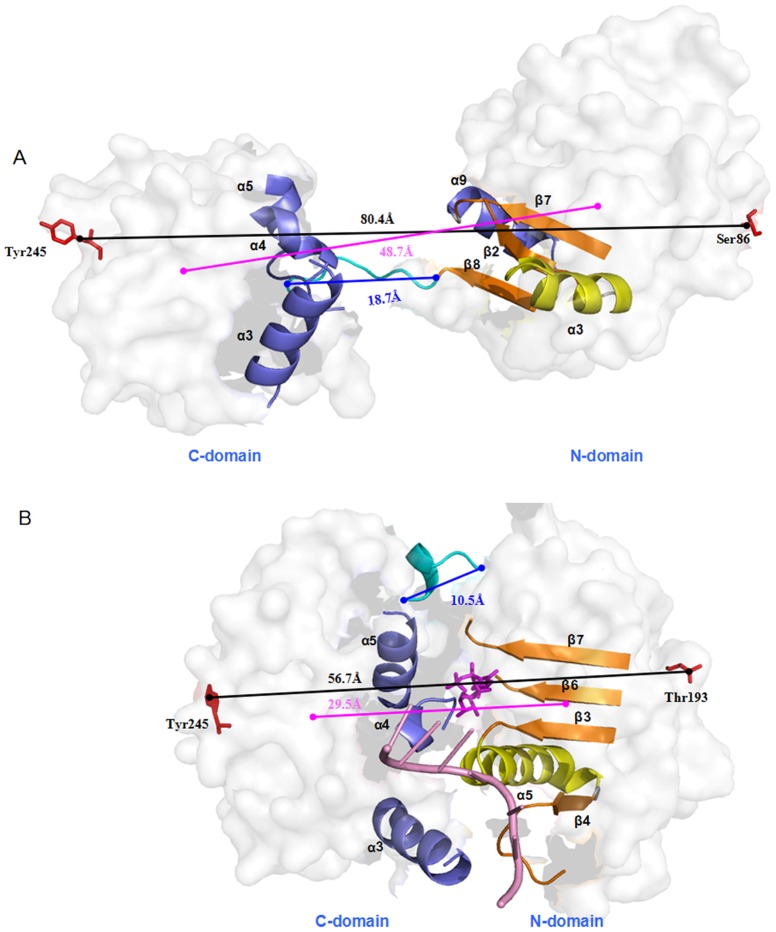
The distances in the open and closed eIF4A states. The length of whole eIF4A protein (black line), the mass center distance between two domains (magenta line) and the length of the linker (blue line) for (A) the open eIF4A state for the O-eIF4A model and (B) the closed eIF4A state in the ATP+RNA+C-eIF4A model; the α helices and β strands at the N-domain – C-domain interface are labeled and shown in cartoon form with the remaining part of the protein colored in white semi-transparent surface; the ATP and RNA molecules are shown by magenta stick and pink cartoon, respectively, in the closed state.

**Figure 6:**
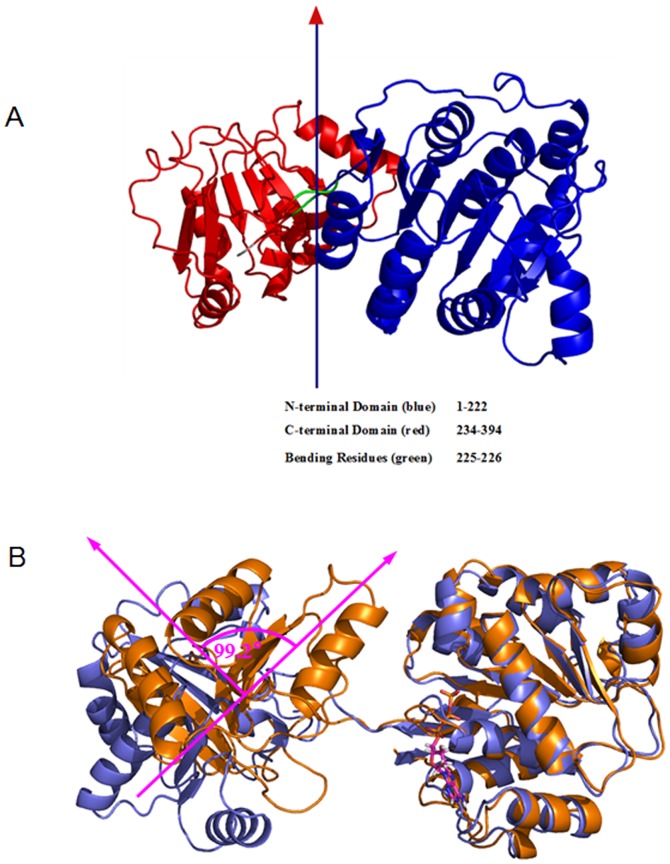
Domain rotation angel between the O-eIF4A and ATP+eIF4A models. (A) Dynamic domain identified between the O-eIF4A and ATP+eIF4A models. The residues forming the fixed domain, moving domain and bending residues are depicted in colors of blue, red and green, respectively, with a reference line crossing at the center of rotation. (B) The relative rotation angel of the two domains in the eIF4A protein between the O-eIF4A (slate) and ATP+eIF4A (orange) models.

Based on the RMSD values of O-eIF4A and ATP+RNA+C-eIF4A models, we obtained two stable states of open eIF4A protein and closed eIF4A protein bound by ATP and RNA molecules. The corresponding average structures of these two systems extracted from the trajectories of their simulations were analyzed and are shown in Figure 3A and B. The eIF4A protein in each conformation state consists of two domains (N- and C- domains), i.e., the N-domain formed by a 5α-8β-4α fold (α_1-5_-β_1-8-2-7-6-3-5-4_-α_6-9_) and the C-domain formed by a 2α-7β-4α fold (α_1-2_-β_3-4-2-5-6-1-7_-α_3-6_) (see Figure 4A and B). The RMSD values less than 4.5 Å in these two models suggest that the average structures of open eIF4A protein and closed ATP+RNA+eIF4A complex primarily represent the characteristics in X-ray crystal structure and homology modeling structure (see Figure 3B), respectively. The most remarkable differences between the O-eIF4A and ATP+RNA+C-eIF4A states are as follows: (1) In the O-eIF4A state, the N- and C-domains separate from each other with their mass center distance of 48.7 Å, and their end-to-end length of 80.4 Å between Cα atoms of Ser86 and Tyr245 residues, and the loop linker length of 18.7 Å between Cα atoms of Val223 and Thr229 residues. The end-to-end and loop linker lengths are consistent with the experimental results of 80 Å and 18 Å, respectively [29]. In the ATP+RNA+C-eIF4A state, such distances decrease to 29.5 Å, 56.7 Å and 10.5 Å, respectively (see Figure 5A and B). (2) The angle changes between the domains of O-eIF4A and ATP+RNA+C-eIF4A models from the simulations have been analyzed by using the DynDom program. The relative rotation angle of two domains in these two states was 103.6° and the corresponding domain decomposition and the hinge-bending residues are shown in Figure 6A. (3) Such proximity and relative rotation of two domains in the two states result in structural changes of the N-domain – C-domain interface. In the O-eIF4A state, the β2/7/8 strands and α3, α9 helices in the N-domain face the α3/4/5 helices in the C-domain, without any interdomain contacts. In contrast, in the ATP+RNA+C-eIF4A state, the α5-β4 segments and β3/6/7 strands in the N-domain face respectively the α3 and α4/5 helices in the C-domain with extensive contacts in the N-domain – C-domain interface (see Figure 5A and B). The interhelical angle of α5 helix in the N-domain and α3 helix in the C-domain, measured with the INTERHLX program, changed from the average value of 97.7° in the O-eIF4A state, without any interaction between the two helices, to that of 65.7° in the ATP+RNA+C-eIF4A one, with some interactions between the two helices. The β3 and β6/7 strands in the N-domain are arranged in parallel, and their C-terminus respectively point to the middle position of α4 and α5 helices in C-domain with the interactions between the β3/6/7 strands and the α4/5 helices in the ATP+RNA+C-eIF4A state. Further, such relative rotation of two domains also causes rearrangement of the RNA binding residues, Arg99, Arg148, and others in the N-domain and Arg269, Arg298, and others, in the C-domain to a linear structure at the edge of eIF4A protein in the ATP+RNA+C-eIF4A state. (4) In the O-eIF4A state, the C-terminus of β2–β8 strands in the N-domain and the C-terminus of β1–β6 strands in the C-domain form an acute angle of ∼60°. In the ATP+RNA+C-eIF4A state, the acute angle changes to ∼90°, i.e. two β-strand groups are almost perpendicular to each other. These results indicate that the significant structural differences involve variations of distance and angle between the N- and C- domains in the two states, which results in the structural change of the N-domain – C-domain interface.

### 2. Free energy calculations revealed that the open-to-closed allosteric process is caused by cooperative binding of ATP or RNA with eIF4A protein

To determine the favorable binding order and cooperativity of ATP and RNA substrate binding to the eIF4A protein, we performed binding free energy calculations of the ATP+eIF4A, RNA+eIF4A and ATP+RNA+C-eIF4A models by using the MM-PBSA methodology based on the MD simulations. All energy terms and the total binding free energies of these systems are displayed in Table 1. It is apparent that the binding free energies of ATP substrate and RNA molecule to the eIF4A protein in the ATP+eIF4A and RNA+eIF4A models, respectively, are −8.62 and −12.57 kcal·mol^−1^, which indicates that the initial binding of ATP substrate or the RNA molecule to eIF4A protein was energetically favorable and allosterically regulated. It is also apparent that the binding free energies of ATP substrate and RNA molecule to the eIF4A protein in the ATP+RNA+C-eIF4A model, respectively, were −30.32 and −64.85 kcal·mol^−1^. It indicates that the binding free energies of ATP substrate and RNA molecule to the eIF4A protein in the ternary systems increased respectively by −21.70 and −52.28 kcal·mol^−1^ compared with the corresponding binary systems, leading to positive cooperative binding with each other for the two substrates. The calculated data support the experimental observation that the affinity of the eIF4A protein for RNA or ATP was enhanced by the coupling binding of ATP or RNA [30]. ATP binding to the eIF4A protein enhances the cooperative binding of the RNA molecule. On the other hand, the present binding energy calculations for the ATP+RNA+C-eIF4A model predict that the closed eIF4A state bound by the two substrates was more energetically stable than the open eIF4A state.

**Table 1:** MM-PBSA free energy (kcal·mol^−1^) components for the ATP+eIF4A, RNA+eIF4A and ATP+RNA+C-eIF4A models.

	ATP+eIF4A	ATP+RNA+C-eIF4A	RNA+eIF4A	ATP+RNA+C-eIF4A
Receptor	eIF4A	eIF4A	eIF4A	eIF4A
Ligand	ATP	ATP	RNA	RNA
ΔE_ele_	−165.13	−171.14	891.80	−52.60
ΔE_vdw_	−27.02	−41.38	−91.55	−80.21
Δ*E*_int_	0.00	8.96	−6.31	−3.07
ΔG_np/solv_	−5.40	−5.19	−9.55	−8.43
ΔG_pb/solv_	159.42	161.67	−844.45	42.15
ΔG_np_	−32.42	−46.57	−101.10	−88.64
ΔG_pb_	−5.71	−9.47	47.35	−10.46
ΔTS	−29.52	−16.72	−49.44	−37.31
ΔH_binding_	−38.14	−47.04	−60.07	−102.16
ΔG_binding_	−8.62	−30.32	−12.57	−64.85

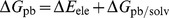
 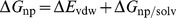 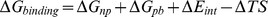 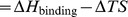

### 3. Characteristics of the ATP and RNA sequential binding to eIF4A protein

#### 3.1. Interaction analysis supports the RNA-mediated positive cooperativity of ATP binding with eIF4A protein

To address the positive cooperativity of RNA for ATP binding with the eIF4A protein, the percentages of occurrences of hydrogen bonds in the ATP+eIF4A and ATP+RNA+C-eIF4A models were analyzed and respectively shown in Table 2 for the ATP–eIF4A interface, in Table 3 and 4 for the RNA–eIF4A interface, and in Table 5 for the N-domain – C-domain interface. The criteria for the intermolecular or intramolecular hydrogen bond included a donor-acceptor distance of <3.5 Å and a donor-proton-acceptor angle of >120° [57], [58]. In the ATP+eIF4A model involving the ATP–eIF4A interface, the total hydrogen bond occupancies of 1328.46% occur apparently around the ATP-binding region at the N-domain of eIF4A protein, i.e., around α2–α3 and β2-P-loop-α4 segments. P-loop is the linker of β2 strand and α4 helix. This interaction analysis supports the binding free energy calculations as discussed above. For the same ATP–eIF4A interface in the ATP+RNA+C-eIF4A model, the hydrogen bond occupancies between the ATP-binding α2–α3/β2-P-loop-α4 region of the N-domain, the ATP-binding α4-β5/α5-β6 region of the C-domain in the eIF4A protein and the ATP substrate increased to 1703.61% compared with the same interface in ATP+eIF4A model, which indicated that ATP interacting with one N-domain in the ATP+eIF4A model bound with both N- and C-domains in the ATP+RNA+C-eIF4A model. For example, additional interaction residues Asp324, Arg349, and Arg352 at the ATP-binding α4-β5/α5-β6 region of C-domain were found (see Table 2). The binding residues reproduced the previous experimental observation [19]. The results predict the tremendous positive cooperativity for ATP binding with eIF4A protein induced by the subsequent RNA binding. Furthermore, in the ATP+RNA+C-eIF4A model, the existence of hydrogen bonds between the RNA binding region at the N-domain, i.e., around β3-α5, β4-β5-α6 segments, the RNA binding region at the C-domain, i.e., around α2, β3-α3, β4-α4 segments in the eIF4A protein and the RNA molecule with the total percentage of 2123.08% (see Table 3 and 4), results in the positive cooperativity for ATP binding mediated structurally by ATP–eIF4A interface. The RNA binding residues Pro97, Arg99, Gly125, Gly126, Thr145, Gly147, Arg148, Arg269, Ser291, Arg298 and Thr316 reproduced the previous experimental observations [19]. On the other hand, we found specific hydrogen bond interactions at the N-domain – C-domain interface, i.e., the α5-β4 and β3/6/7 segments of the N-domain versus the α3 helix and α4/5 helices of the C-domain in the ATP+RNA+C-eIF4A model, respectively, of which β3, β4, α5 of N-domain and α3, α4 of C-domain not only belong to RNA-binding region but also belong to N-domain – C-domain interface (see Table 5). However, such interactions were not found in the ATP+eIF4A model. Specific interaction residues in the N-domain – C-domain interface also reproduced the previous experimental observations [19].

**Table 2:** The occupancies (%) of hydrogen bonds between the ATP and eIF4A protein for the ATP+eIF4A and ATP+RNA+C-eIF4A models.

		Hydrogen bond	ATP+eIF4A	ATP+RNA+C-eIF4A
N-domain	α2–α3	(ATP)N6-H…O(Gln43)	98.61	70.56
		(ATP) N7-H…OG (Ser45)	97.95	0
		(ATP) N7-H…NE2 (Gln48)	35.17	68.78
		(ATP)N6-H…OE1(Gln48)	99.88	73.42
	P-loop	(ATP) O1A-H…N (Gln66)	88.99	0
		(ATP) O1A-H…N (Ser67)	99.66	0
		(ATP) O1G-H…OG (Ser67)	71.64	99.32
		(ATP) O1B-H…N (Gly68)	0	53.56
		(ATP) O3B-H…N (Gly68)	76.70	98.72
		(ATP) O1G-H…N (Gly68)	59.38	12.10
		(ATP)O3′-H…OG1(Thr69)	52.80	0
		(ATP) O1B-H…N (Thr69)	0	67.26
		(ATP) O1B-H…N (Gly70)	0	98.88
		(ATP) O5′-H…N (Gly70)	92.56	0
		(ATP) O3A-H…N (Gly70)	90.49	30.62
	α4	(ATP) O1B-H…N (Lys71)	0	98.08
		(ATP) O2B-H…N (Lys71)	95.75	4.48
		(ATP) O2A-H…N (Lys71)	62.41	0
		(ATP) O1G-H…NZ (Lys71)	77.65	99.70
		(ATP) O2B-H…NZ (Lys71)	75.00	97.76
		(ATP) O3G-H…NZ (Lys71)	53.82	0.60
		(ATP) O2B-H…N (Thr72)	0	73.56
		(ATP) O1A-H…N (Thr72)	0	42.54
		(ATP) O1A-H…N (Gly73)	0	98.85
C-domain	α4-β5	(ATP)O3′-H…OD1(Asp324)	0	44.88
	α5	(ATP) O2G-H…NH1 (Arg349)	0	99.96
		(ATP) O2G-H…NH2 (Arg349)	0	99.60
		(ATP) O1G-H…NH1 (Arg349)	0	86.74
	α5-β6	(ATP) O2G-H…NH1 (Arg352)	0	97.80
		(ATP) O2G-H…NH2 (Arg352)	0	85.84

**Table 3:** The occupancies (%) of hydrogen bonds between the RNA molecule and the N-domain (ND) of the eIF4A protein for the I, II, III, IV and ATP+RNA+C-eIF4A (C) models.

ND	Hydrogen bond	I	II	III	Hydrogen bond	IV	C
β3-α5	(U402)O2′…O-H(Pro97)	10.12			(U400)O2′…O-H(Pro97)		80.12
	(U402)O2-H…N(Arg99)	16.62	50.16		(U401) O1P-H…OG1(Thr98)		96.54
	(U403)O1P-H…NE(Arg99)			6.62	(U402) O1P-H…NE(Arg99)		21.70
	(U403)O1P-H…NH2(Arg99)			20.98	(U402)O2P-H…NH2(Arg99)		99.18
	(U403)O2′-H…NE(Arg99)		11.90		(U401) O1P-H…N(Arg99)	98.51	99.78
	(U402)O2′-H…N(Arg99)	33.12			(U402) O2P-H…NE(Arg99)		98.40
β4-β5-α6	(U404)O2-H…N(Gly126)		20.52		(U402)O1P-H…N(Gly125)		82.06
	(U404)O4′-H…N(Gly126)		21.82		(U403)O1P-H…N(Gly126)		99.82
	(U401) O4-H…OG1(Thr145)		13.24		(U402)O1P-H…OG1(Thr145)	99.91	100.0
	(U402) N3-H…OG1(Thr145)		25.80		(U401) O2′-H…N(Gly147)	54.71	40.46
	(U401) O4-H…N(Gly147)		92.04	29.28	(U402)O2′-H…NE(Arg148)		69.26
	(U402) O2-H…NH1(Arg148)			98.26	(U402)O1P-H…N(Arg148)		64.10
	(U401) O4-H…N(Arg148)		11.88		(U402) O3′-H…NE(Arg148)		43.26
	(U402) O4-H…NH2(Arg148)		10.30		(U402)P-H…NH1(Arg148)	37.22	
	(U402) O4-H…NE(Arg148)		31.62		(U403)O1P-H…NH2(Arg148)	11.98	97.66
	(U404)O1P-H…NH2(Arg148)	6.42			(U402)O1P-H…NH1(Arg148)	94.99	
	(U402)N3-H…OD2(Arg151)			76.54	(U402)O2′-H…OD1(Arg151)		33.28
α7-α8	(U399)N3-H…OE2(Glu173)			67.76			
	(U400)N3-H…OE1(Glu173)	42.54					
	(U400) O1P…H-OG(Ser177)		85.16				
	(U399) O2′-H…O(Ser177)		62.94				
	(U400) O2-H…NE2(Gln182)		81.50				

**Table 4:** The occupancies (%) of hydrogen bonds between the RNA molecule and the C-domain (CD) of the eIF4A protein for the I, II, III, IV and ATP+RNA+C-eIF4A (C) models.

CD	Hydrogen bond	I	II	III	Hydrogen bond	IV	C
α2	(U396)O2′…H-OD1(Pro267)	51.12			(U398)O2′…H-O(Pro267)	85.60	66.50
	(U396)O2-H…ND2 (Asn267)	46.98			(U399)O1P-H…N(Arg269)	100.0	94.72
	(U397) P-H…NH1(Arg269)		10.5		(U399) P-H…NE(Arg269)	56.92	9.70
	(U398)O2P-H…NH1(Arg269)		29.30		(U399)O2P-H…NH2(Arg269)	99.51	50.30
	(U397)O2P-H…NH2(Arg269)		21.94		(U399)O5′-H…NE(Arg269)	64.19	9.66
	(U398)O1P-H…NH1(Arg269)			14.00	(U399)O5′-H…NH2(Arg269)	32.78	
	(U397) O5′-H…NH1(Arg269)		22.62		(U399)O1P-H…NE(Arg269)	98.31	36.72
	(U397) O1P-H…N(Arg270)		13.42		(U399) P-H…NH2(Arg269)	39.13	
β3-α3	(U398) O1P-H…OG(Ser291)	32.58		99.82	(U400)O1P-H…OG(Ser291)	6.73	21.88
	(U398)O2P-H…OG(Ser291)	79.70	71.30		(U400) P-H…OG(Ser291)	60.00	61.46
	(U399)O2P-H…OG(Ser291)		23.22		(U400)O2P-H…OG(Ser291)	99.98	99.34
	(U398)O1P-H…N(Ser291)	73.08			(U400)O1P-H…N(Ser291)	99.25	100.0
	(U398) P-H…OG(Ser291)	59.44	24.18				
	(U400)O2P-H…NE2(Gln295)			64.80	(U402)O2P-H…NE2(Gln295)	98.59	
	(U398) O3′-H…NH1(Arg298)	69.20			(U401)O2P-H…NE2(Gln295)	97.27	
	(U399)O1P-H…NH2(Arg298)	89.14	54.24	52.96	(U401)O1P-H…NH2(Arg298)		99.76
	(U399)O1P-H…NH1(Arg298)	44.44	92.62	82.48	(U401)O2P-H…NH1(Arg298)	99.95	99.02
	(U399)O2P-H…NH2(Arg298)		92.70	16.66	(U401)O2P-H…NH2(Arg298)	17.71	52.42
β4-α4	(U397)O3′-H…OG1(Thr316)	23.08			(U399)O3′-H…OG1(Thr316)		58.88
	(U398) P-H…OG1(Thr316)	49.52			(U400) P-H…OG1(Thr316)		41.86
	(U398)O1P-H…OG1(Thr316)	90.20			(U400)O1P-H…OG1(Thr316)		95.24
	(U397)O2′…H-OG1(Thr316)	31.94					
	(U397) O2-H…N(Leu318)	94.26					

**Table 5:** The occupancies (%) of hydrogen bonds of the N-domain – C-domain interface in the ATP+eIF4A (ATP+4A), I, II, III, IV and ATP+RNA+C-eIF4A (C) models.

Hydrogen bond	ATP+4A	I	II	III	IV	C
(99Arg) N-H…O(295Gln)	0	0	0	1.62	32.75	99.82
(100Glu) O-H…N(296Gln)	0	0	37.22	0	0	0
(100Glu) O-H…N(298Arg)	0	0	0	0	0	207.16
(100Glu) O-H…N(306Arg)	0	0	0	222.74	123.94	0
(104Gln) O-H…N(324Asp)	0	0	0	0	99.57	115.14
(104Gln) N-H…N(324Asp)	0	0	0	0	9.87	15.28
(104Gln) N-H…O(324Asp)	0	0	0	28.78	99.73	0
(104Gln) N-H…O(322Gly)	0	0	0	0	0	45.58
(124Ile) O-H…N(295Gln)	0	0	0	0	33.80	0
(170Glu) O-H…N(321Arg)	0	0	0	0	0	99.50
(170Glu) O-H…N(345Hie)	0	0	0	0	0	135.38
(172Asp) O-H…N(321Arg)	0	0	0	0	2.87	288.08
(172Asp) O-H…N(345Hie)	0	0	0	0	39.08	0
(173Glu) O-H…N(321Arg)	0	0	7.96	286.88	169.89	183.18
(200Ser) O-H…N(321Arg)	0	0	0	0	0	89.06
(202Thr)O-H…N(321Arg)	0	0	0	0	0	40.2
(202Thr)O-H…N(345Hie)	0	0	0	0	48.61	0
(202Thr)O-H…N(341Gln)	0	0	0	0	80.24	0

#### 3.2. Allosteric modulation of eIF4A protein induced by ATP and RNA sequential binding

**Figure 7:**
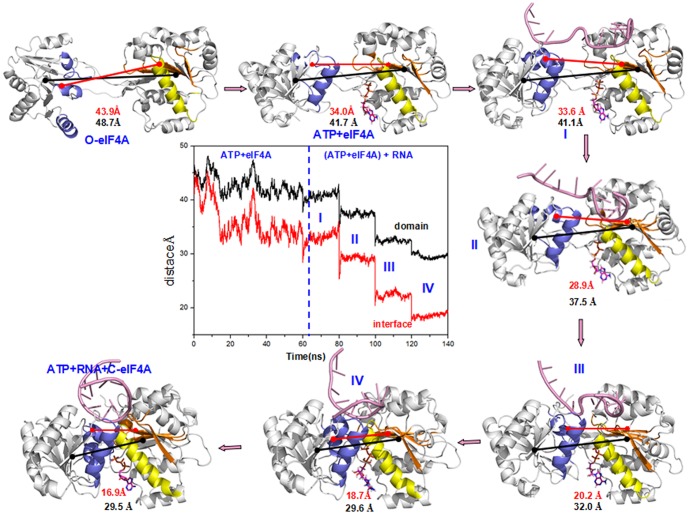
The structures of the allosteric process. The average structures are extracted from the trajectories of the O-eIF4A, ATP+eIF4A, I, II, III, IV and ATP+RNA+C-eIF4A models involved in the allosteric process of the ATP binding followed by RNA, i.e., the ATP+eIF4A model for the ATP first binding to the O-eIF4A model; the I-IV models for the RNA second binding to the equilibrium structure of the ATP+eIF4A model; the ATP+RNA+C-eIF4A model for the closed state of the eIF4A protein. The center image shows the time-dependence of the mass center distance between two domains (black), and that of N-domain – C-domain interface (red) during the simulation.

**Figure 8:**
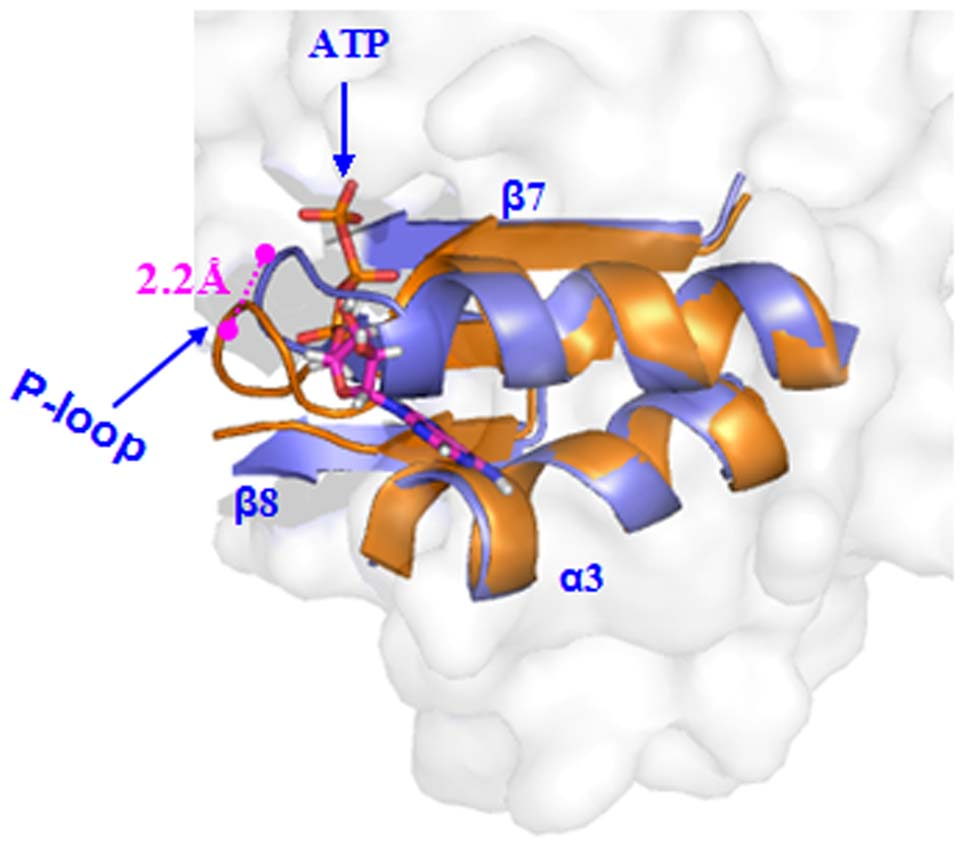
The difference of distances of P-loops. The difference of the mass center distances of the ATP binding P-loops between the O-eIF4A (slate) and ATP+eIF4A (orange) models.

**Figure 9:**
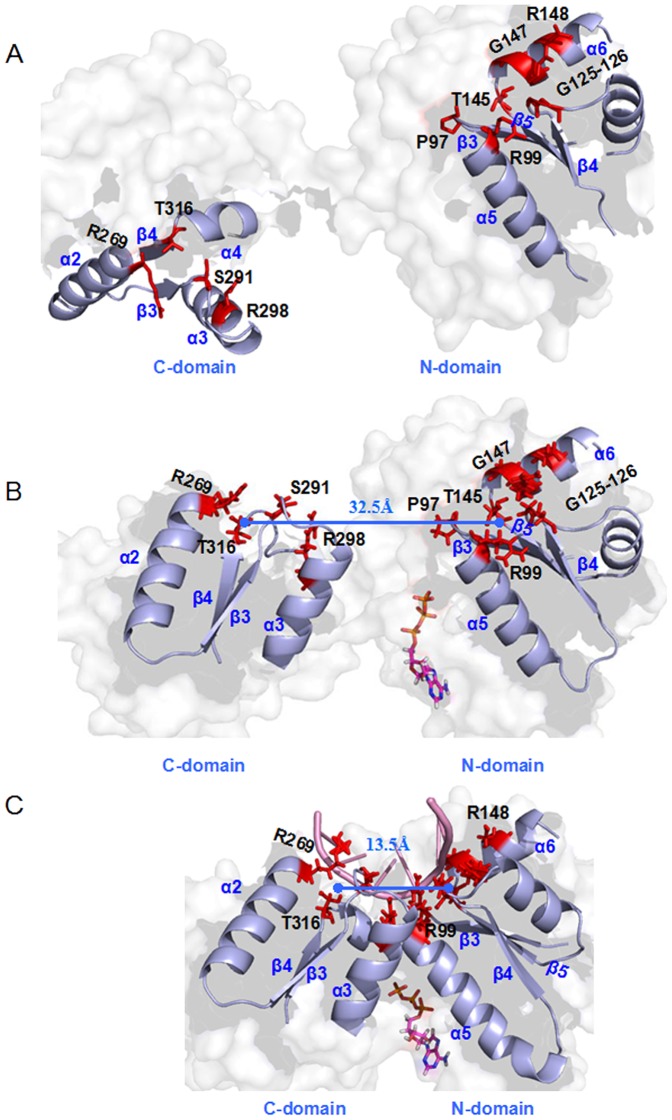
The distances of RNA binding sites in the eIF4A protein. The residues (red sticks) at the RNA binding region (α helices and β strands) in (A) the O-eIF4A, (B) ATP+eIF4A and (C) ATP+RNA+C-eIF4A models with the mass center distances of RNA binding sites at the two domains (light blue line).

Based on the structural differences of eIF4A protein in the O-eIF4A and ATP+RNA+C-eIF4A models, the conformational changes of eIF4A protein induced by the ATP and RNA sequential binding were analyzed by using the allosteric simulation of the O-eIF4A model to the ATP+eIF4A model, as the first step, and four RNA mediating models, I, II, III, IV, initiating from the TMD simulation of (ATP+eIF4A)+RNA→(ATP+RNA+C-eIF4A), as the second step (see Figure 7). These four models, I, II, III, IV were simulated by the CMD method and attained the corresponding stable configurations. The corresponding RMSD values are displayed in Figure S2A–D. The first step entailed ATP-mediated shift in the P-loop away from β7 strand and close to both β8 and α3 segments with ∼2.2 Å movement distance along with the formation of hydrogen bonds at the ATP-binding region (see Figure 8). Such movement of the P-loop results in its open conformation, as reported experimentally [59]. Interestingly, the initial ATP binding causes a rotation of 99.2° between the two domains of protein relative to the O-eIF4A model (see Figure 6B), which results in structural changes of the N-domain – C-domain interface and the RNA-binding regions at the N- and C- domains. At the N-domain – C-domain interface, the α5-β4 and β3/6/7 interface segments in the N-domain gradually move towards the α3 and α4/5 interface helices, respectively, in the C-domain. Simultaneously, the residues Pro97, Thr98, Arg99, Gly125, Gly126, Thr145, Gly147, Arg148 at the RNA-binding β3-α5/β4-β5-α6 region of N-domain and the residues Arg269, Ser291, Arg298, Thr316 at the RNA-binding α2/β3-α3/β4-α4 region of C-domain are rearranged to a linear structure, and are located at the edge of N-/C- domain, which may favor RNA binding to eIF4A protein in the cytoplasm (see Figure 9A and B). In addition, the mass center distance between the two domains was reduced by ∼7 Å due to the ATP binding. The ATP mediation in the first step facilitates the partial open-to-closed transition.

In the second step involving RNA sequential binding, the RNA binding sites at the N-domain close to the C-domain showed a decrease in their mass center distance from 32.5 Å in the ATP+eIF4A model to 13.5 Å in the ATP+RNA+C-eIF4A model (see Figure 9B and C). Such proximity further narrowed the average mass center distances from 41.7 Å in the ATP+eIF4A model, 41.1 Å in the I model, 37.5 Å in the II model, 32.0 Å in the III model, 29.6 Å in the IV model, to 29.5 Å in the ATP+RNA+C-eIF4A model (see Figure 7). As expected, the proximity of two domains induces the gradual formation of hydrogen bonds in the N-domain – C-domain interface as follows: 0% in the I model, 45% in the II model, 540% in the III model, 740% in the IV model, and 1318% in the ATP+RNA+C-eIF4A model (see Table 5). With the proximity of two domains, the binding bases on the RNA molecule simultaneously migrate along the edge of eIF4A protein from the I model with the RNA binding bases U396–U402 to the II model with U397–U404, to the III, IV and ATP+RNA+C-eIF4A models with U398–U403, which was also apparent from the hydrogen bond formations at the RNA–eIF4A interface (see Tables 3 and 4). In brief, the two allosteric steps indicated that the ATP initial binding to the open eIF4A protein causes the relative rotation of two domains, while the RNA binding induces considerable proximity of two domains. The cooperative binding mediated by ATP and RNA completes the open-to-closed transition for the eIF4A protein.

#### 3.3. Dynamic fluctuation and correlation of the transition from O-eIF4A model to the ATP+eIF4A model and from the ATP+eIF4A model to the ATP+RNA+C-eIF4A model

**Figure 10:**
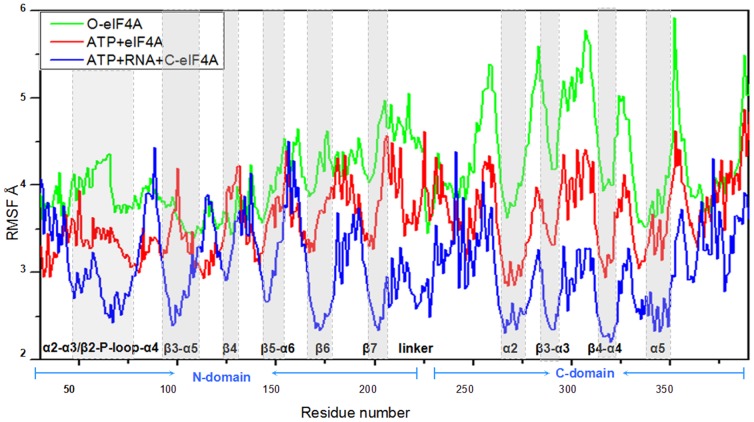
The fluctuations of residues. The fluctuations of residues in the O-eIF4A (green), ATP+eIF4A (red) and ATP+RNA+C-eIF4A (blue) models with the labeled α helices and β strands at the ATP/RNA binding region and the N-domain – C-domain interface in the shaded regions.

To further investigate the interaction between the eIF4A protein and the ATP or RNA molecule and the conformational changes of eIF4A protein induced by the ATP and RNA binding via the residue position changes, the dynamics of every residue was determined and interpreted by residue fluctuations and correlations. The RMSF values of the eIF4A protein in the O-eIF4A, ATP+eIF4A and ATP+RNA+C-eIF4A models were analyzed and shown in Figure 10. The additional hydrogen bonds between the eIF4A protein and ATP substrate in the ATP+eIF4A model with respect to the O-eIF4A, and between the eIF4A protein and RNA in the ATP+RNA+C-eIF4A model with respect to ATP+eIF4A model are directly linked to the changes of RMSF values in the fluctuation pattern. Namely, the newly-formed hydrogen bonds between the residues Gln43, Ser45, Gln48, Gln66-Lys71 at the ATP-binding α2–α3/β2-P-loop-α4 region of the N-domain in the eIF4A protein and the ATP substrate in the ATP+eIF4A model caused the decrease in corresponding fluctuations compared with those in the O-eIF4A model, which contributes to the stabilization of corresponding contact sites. Further, the newly-formed hydrogen bonds between the residues Arg99, Arg148, etc, at the RNA-binding β3-α5/β4-β5-α6 region of the N-domain, Arg269, Ser291, Arg298, etc, at the RNA-binding α2/β3-α3/β4-α4 region of the C-domain in the eIF4A protein, and RNA molecule in the ATP+RNA+C-eIF4A model caused the decrease of corresponding fluctuations compared with those in the ATP+eIF4A model. Furthermore, the fluctuations at the residues of newly-formed hydrogen bonds, between the residues Arg99–Arg100, Glu170–Glu173, etc, in the interface α5-β4/β3/6/7 segments of the N-domain and the residues Gln295, Arg298, Arg321, etc, in the interface α3/4/5 helices of the C-domain considerably decreased in the ATP+RNA+C-eIF4A model compared with those in the O-eIF4A and ATP+eIF4A models. These results are consistent with the hydrogen bond analysis discussed above.

**Figure 11:**
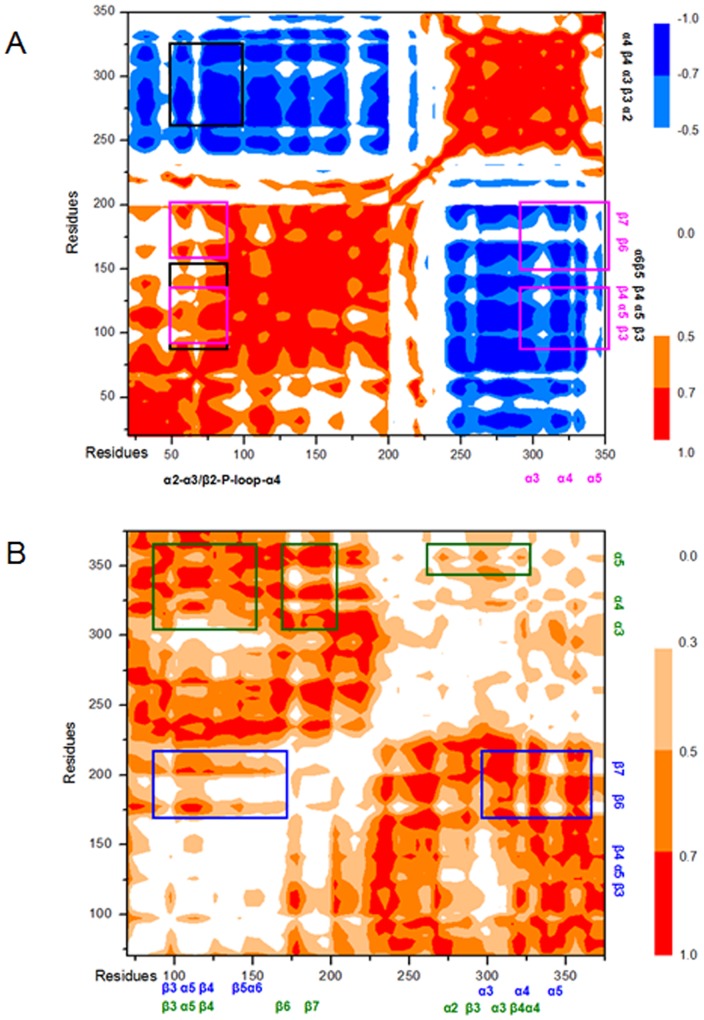
Correlation maps and the differences of correlations. Dynamical cross-correlation maps for (A) the allosteric ATP+eIF4A simulation and (B) the differences of motion correlations between the ATP+RNA+C-eIF4A and ATP+eIF4A equilibrium simulations, with specific sub-regions squared in black for the correlations of the ATP binding and RNA binding regions, in magenta for the correlations of the ATP binding region and the N-domain – C-domain interface, in blue for the correlations of the N-domain RNA binding region and the N-domain – C-domain interface, and in green for the correlations of the C-domain RNA binding region and the N-domain – C-domain interface.

To explore the allosteric communication of positional changes in the ATP/RNA binding regions and the N-domain – C-domain interface in the transition from open to closed conformations, we constructed and analyzed the motion correlations and their differences in all Cα atoms of the eIF4A proteins from the trajectories. They are respectively displayed in Figure 11A for the first step from the O-eIF4A model to the ATP+eIF4A model and in Figure 11B between the equilibrium simulations of the ATP+eIF4A and ATP+RNA+C-eIF4A models. These two maps show the motion correlations between the residues ranging from highly anticorrelated (blue) to highly correlated (red). As illustrated in Figure 11A the motions of ATP-binding α2-α3/β2-P-loop-α4 region in the N-domain, significantly correlate and anticorrelate with the motions of the RNA-binding β3-α5/β4-β5-α6 region of N-domain and the RNA-binding α2/β3-α3/β4-α4 region of C-domain, respectively, (represented by the black squares in Figure 11A), which predicts that the ATP binding causes the allostery of the RNA binding sites in the eIF4A protein, resulting in strong hydrogen bond formation and the high affinity between the protein and RNA. Further, the large correlated motions of ATP-binding region vs the α5-β4/β3/6/7 interface segments of the N-domain occur remarkably with the large anticorrelated motions of this α5-β4/β3/6/7 interface segments vs the α3/4/5 interface helices of the C-domain (represented by the magenta squares in Figure 11A), which predicts that the ATP binding to eIF4A leads to allosteric communication from ATP-binding region to domain-domain interface, resulting in the interface structure changes mediated by the rearrangement of the N- and C-domains with the relative rotation angle of 99°, which completes the partial transition of the eIF4A protein from the open to closed state. As shown in Figure 11B the large correlated motions of the RNA-binding β3-α5/β4-β5-α6 region of N-domain vs the α5-β4/β3/6/7 interface segments of N-domain occur remarkably with the large correlated motions of this α5-β4/β3/6/7 interface segments of N-domain vs the α3/4/5 interface helices of C-domain (see the blue squares in Figure 11B). Similar effect of the RNA-binding α2/β3-α3/β4-α4 region upon the C-domain interface was seen, and further on the N-domain interface (see the green squares in Figure 11B). It indicates that the RNA binding with eIF4A leads to allosteric communication from RNA-binding region to domain-domain interface, resulting in proximity between N- and C-domains, and the final transition of the eIF4A protein from the open to closed state.

### 4. Characteristics of the RNA and ATP sequential binding to eIF4A protein

To address the allosteric mechanism involving the sequential binding of RNA and ATP, the RNA+eIF4A model of the RNA initial binding with the eIF4A protein and four I', II', III', IV' models (involved in the ATP second binding to the RNA+eIF4A model in the allosteric step of (RNA+eIF4A)+ATP→(ATP+RNA+C-eIF4A)) were simulated. The corresponding results are shown in Tables S1, S2, S3 and Figures S2E–H, S3, S4, S5, S6. Based on the binding free energy calculations discussed above, the ATP binding to the RNA+eIF4A complex also enhances the cooperative binding of RNA with eIF4A protein. As shown in Tables S1 and S2 involving the RNA–eIF4A interface in the RNA+eIF4A model, the total hydrogen bond occupancies of 1452.08% occur apparently between the RNA-binding β3-α5/β4-β5-α6 region of N-domain, the RNA-binding α2/β3-α3/β4-α4 region of C-domain and the U395–U397, U404–U406 bases of the RNA molecule. We found 694.12% additional occupancies for such interface in the ATP+RNA+C-eIF4A model, involving the extra hydrogen bonds between the Gly126, Ser291, Arg298, Thr316 residues at the RNA-binding regions and U399, U400, U401, U403 bases of RNA molecule. It also indicated the great positive cooperativity of RNA binding with eIF4A protein induced by ATP binding. Furthermore, the conformational changes of eIF4A protein induced by the initial RNA and later ATP binding have also been analyzed and are shown in Figure S3. The first allosteric step involves the mechanism of RNA mediating eIF4A protein. The second allosteric step involves the ATP binding to the RNA+eIF4A model through four mediating models of I', II', III', IV'. In the first step, RNA binding to eIF4A protein causes the proximity of the two domains by decreasing their average mass center distances from 48.7 Å in the O-eIF4A model to 38.2 Å in the RNA+eIF4A model, resulting in the structural changes and hydrogen bond formation at the N-domain – C-domain interface (see Table S3). The convex structure of RNA molecule around the bases U398–U403 at the edge of eIF4A protein was formed during this step (see Figure S3). In the second step, the ATP binding causes relative rotation of the two domains in the eIF4A protein with 28° in I' model, 48° in II'/III' model, and 63° in the IV'/ATP+RNA+C-eIF4A model, relative to the RNA+eIF4A model (see Figure S4), and the migration of the RNA-binding bases along the edge of eIF4A protein. The original convex structure (I', II') of RNA molecule gradually transforms into a linear structure (III'), and finally to a concave structure (IV') with the migration of RNA-binding bases from the original bases U395–U397, U404–U406 to new binding bases U398–U403 mediated by ATP binding (see Figure S3 and Table S1 and S2). Simultaneously, the cooperative mediation by ATP and RNA induces the proximity of the two domains with the decrease in their average mass center distances from 38.1 Å in the RNA+eIF4A/I' model, 35.5 Å in the II' model, 32.5 Å in the III' model, to 29.8 Å in the IV'/ATP+RNA+C-eIF4A model to form the final closed state. The cooperative occurrence of such relative rotation and proximity of the two domains induced by ATP and RNA binding to the eIF4A protein suggests that the cooperative mediation of ATP and RNA plays a key role in the structural transition of eIF4A protein from the open to closed state, which supports previous results reported by Klostermeier and co-workers [32], [34]. As expected, the hydrogen bond strength at the N-domain – C-domain interface gradually increases from the RNA+eIF4A model to I', II', III', IV' and the ATP+RNA+C-eIF4A models induced by such cooperative mediation (see Table S3). Similar findings for the RMSF and correlation motion analysis are illustrated in Figure S5 and Figure S6A and B. In summary, the two allosteric steps indicated that initial RNA binding to the open eIF4A protein induces the partial proximity of the two domains, while ATP binding causes relative rotation of the two domains and migration of RNA binding bases, and facilitates RNA-mediated proximity of the two domains, finally forming the closed eIF4A conformation.

Interestingly, ATP binding to the eIF4A protein caused the rotation of two domains and the RNA base migration, regardless of the order of binding. However, RNA mediating the proximity of two domains via the migration of RNA bases depends partially on ATP binding. Therefore, the ATP and RNA cooperative binding completes the conformational transition of the eIF4A protein from the open to closed state. Furthermore, to compare the intermediate structures of the eIF4A protein in the present allosteric processes with the X-ray structure of the eIF4G-eIF4A complex as “half-open” conformation, the mass center distances and the relative angles of rotation were analyzed with respect to the open state between two domains as shown in Figure S7. The corresponding distance of 38.3 Å in the eIF4G-eIF4A complex approximates the values of 37.5 Å for the II model and 38.0 Å for the I' model as the intermediates induced by the RNA mediating in the two allosteric processes. Similarly, the rotation angle of 101° in the eIF4G-eIF4A complex is quite close to the average values of 106° for the II model and 99° for the I' model induced by the ATP mediating.

## Discussion

### Allosteric network induced by ATP binding

We explored the relationships between the motion correlations and their structural changes in order to understand fully the allosteric communication network of the eIF4A protein from the ATP binding region to the RNA binding region and the N-domain – C-domain interface for the two allosteric processes mediated by the ATP substrate. Binding with ATP initially followed by RNA showed that the motions of ATP-binding α2-α3/β2-P-loop-α4 region (i.e., around the Gln43, Ser45, Gln48, Gln66, Ser67, Gly68, Thr69, Gly70 and Lys71 residues), correlate and anticorrelate with the motions of the RNA-binding β3-α5/β4-β5-α6 region of N-domain (i.e., around Pro97, Thr98, Arg99, Gly125, Gly126, Thr145, Gly147, Arg148 residues) and RNA-binding α2/β3-α3/β4-α4 region of C-domain (i.e., around Arg269, Ser291, Arg298, Thr316 residues), respectively (see Figure 11A). As expected, the structural change relative to this correlation involves that the structural variation of ATP-binding region with the movement of P-loop relative to β7/8/α3 segments and the hydrogen bond formation at the ATP–eIF4A interface communicate to the RNA-binding region of N-domain and C-domain. It entailed rearrangement of residues at the RNA-binding β3-α5/β4-β5-α6 region of N-domain and the RNA-binding α2/β3-α3/β4-α4 region of C-domain. Due to the structural changes in the ATP-binding region, we found that the hydrogen bond formation between the O-H group of Gln48, the N-H groups of Gly68/Lys71 in N-domain and the N/O atoms of ATP molecule yielded occupancies of 99%, 76% and 77% of simulation times, respectively. The RNA binding residues, Pro97, Thr98, Arg99, Gly125, Gly126, Thr145, Gly147, Arg148, Arg269, Ser291, Arg298 and Thr316, rearranged into a linear structure located at the edge of N- and C-domains, which favored the RNA binding to eIF4A with hydrogen bond formation. The motions of ATP-binding α2-α3/β2-P-loop-α4 region also correlated with the motions of the α5-β4/β3/6/7 segments of the N-domain at the N-domain – C-domain interface. Further, the motions of such segments of the N-domain correlated with the motions of the α3/4/5 interface helices of the C-domain. As expected, such correlations predicted that the movement of P-loop away from the β7 strand and close to both β8 and α3 segments at the ATP-binding region of N-domain corresponded to the movements of the α5-β4 and β3/6/7 segments of the N-domain towards the α3 and α4/5 helices of the C-domain, respectively, at the N-domain – C-domain interface, leading to a 99.2° relative rotation of two domains.

In case of the initial RNA binding followed by ATP, it was apparent (see black squares in Figure S6B) from the differences in motion correlation between the equilibrium simulations of the RNA+eIF4A and the ATP+RNA+C-eIF4A models that the motions of ATP-binding α2-α3/β2-P-loop-α4 region (i.e., around the Gln43, Ser45, Gln48, Gln66, Ser67, Gly68, Thr69, Gly70 and Lys71 residues) significantly correlate with the motions of RNA bases (i.e., U395–U406). As expected, the structural changes relative to such correlation involve that a variation of the ATP-binding P-loop communicates to the migration of the RNA-binding bases U395–U406, leading to RNA structural changes with the additional hydrogen bond formation between the RNA molecule and the eIF4A protein. The P-loop first elongates in the I' model, shortens in the II', and moves away from the β7 strand and close to both β8 and α3 segments at N-domain in the III', IV' and ATP+RNA+C-eIF4A models. The original convex structure of RNA molecule involving the RNA binding bases U395–U397, U404–U406 in the I' model slightly changes to a small convex structure with the RNA binding bases U398, U400–U406 in the II' model, and extends to a linear structure with the RNA binding bases U397–U403, U406 in the III' model, finally forming a concave structure with the binding bases U398–U403 in the IV' and ATP+RNA+C-eIF4A models (see Figure S3, Table S1 and S2). It suggests that the RNA binding sites gradually migrate from the ends of RNA sequence to its center, resulting probably in the unwinding of RNA duplex [34]. In addition, such migration of the RNA bases is accompanied by 694% increase in hydrogen bond formation at the RNA–eIF4A interface in the ATP+RNA+C-eIF4A model compared with the RNA+eIF4A model. For example, we found extra hydrogen bonds between N-H groups of Gly126, Arg298 and O atoms of RNA phosphate backbone at the bases U403, U401 with occupancies of 99.8% and 244.0% of simulation times, respectively. These observations suggested the positive cooperativity of the RNA binding to the eIF4A protein induced by the ATP second successive binding. In addition, the motion of ATP-binding α2-α3/β2-P-loop-α4 region also correlated with the motion of the α5-β4/β3/6/7 interface segments of N-domain. Such segmental motion in N-domain further correlated with the motions of the α3/4/5 interface helices in C-domain (see magenta squares in Figure S6B) due to initial ATP binding to the protein at N-domain. As expected, such correlation network indicates that the movement of ATP-binding region structurally communicates to the N-domain – C-domain interface, with the α5-β4 and β3/6/7 interface segments of the N-domain gradually and respectively moving towards the α3 and 4/5 interface helices of the C-domain, resulting in the relative rotation and proximity of the two domains by ∼60° and ∼11 Å, respectively. The α5-β4 and β3/6/7 segments of the N-domain show a relative rotation and proximity respectively with the α3 and 4/5 helices of the C-domain, resulting in increased hydrogen bond formation in the N-domain – C-domain interface. For example, we found the hydrogen bond formation with the total occupancies of 54% in the RNA+eIF4A model, 51% in the I' model, 127% in the II' model, 179% in the III' model, 589% in the IV' model, 1318% in the ATP+RNA+C-eIF4A model (see Table S3).

In summary, the ATP allosteric network analysis demonstrates that the ATP binding with eIF4A protein mediated the relative rotation of two protein domains and the rearrangement of RNA binding sites at the edge of protein, which favored the RNA binding to the eIF4A protein, and especially promoted the RNA-mediated proximity of the two domains via RNA binding base migration.

### Allosteric network induced by RNA binding

To understand fully the allosteric communication network in the RNA-mediated eIF4A binding from the RNA binding region to the N-domain – C-domain interface in the two allosteric processes, we explored the relationships between the motion correlations and their structural changes. Binding with ATP first followed by RNA yielded differences in motion correlations between the equilibrium simulations of the ATP+eIF4A model and the ATP+RNA+C-eIF4A model. The motions of RNA-binding β3-α5/β4-β5-α6 region of N-domain (i.e., around the RNA binding residues Pro97, Arg99, Gly125, Gly126, Thr145, Gly147, Arg148), correlated with the motions of the α5-β4/β3/6/7 interface segments of the N-domain. Such motions of the interface segments correlated with the motions of the α3/4/5 interface helices of the C-domain. Similar correlation occurred involving RNA-binding α2/β3-α3/β4-α4 region, C-domain and N-domain interfaces (see Figure 11B). As expected, the structural variation of RNA-binding region reflected the proximity between the RNA-binding regions of N- and C-domains and the hydrogen bond formation at the RNA-eIF4A interface. The proximity of the RNA-binding regions communicated to the movement of α5-β4 and β3/6/7 interface segments at N-domain towards the α3 and α4/5 interface helices at C-domain, respectively, and the new hydrogen bond formations in the N-domain – C-domain interface in the I, II, III and IV models. In detail, the RNA binding residues Pro97, Thr98, Arg99, Gly125, Gly126, Thr145, Gly147, Arg148 at RNA-binding α5-β4/β3/6/7 region of N-domain moved close to the RNA binding residues Arg269, Ser291, Arg298, Thr316 at RNA-binding α2/β3-α3/β4-α4 region of C-domain. The decreased mass center distance from 32.5 Å in the ATP+eIF4A model, 31.4 Å in the I model, 24.0 Å in the II model, 18.9 Å in the III model, 14.5 Å in the IV model, to 13.5 Å in the ATP+RNA+C-eIF4A model, suggested the proximity between the RNA-binding region of N-domain and that of C-domain. Such proximity accompanied the hydrogen bond formation at the RNA–eIF4A interface with the average total occupancies of 309% and 540% at N-domain and C-domain, respectively, for the first three models (I, II and III) and of 761% and 1077% for the IV and ATP+RNA+C-eIF4A models (see Tables 3 and 4), which predicts the increase of affinity between eIF4A protein and RNA molecule. Furthermore, such proximity of N- and C-domains was associated with the gradual decrease in average mass center distance of the interface segments at N- and C-domains from 34.0 Å in the ATP+eIF4A model, 33.6 Å in the I model, 28.9 Å in the II model, 20.2 Å in the III model, 18.0 Å in the IV model, to 16.9 Å in the ATP+RNA+C-eIF4A model. It was followed by the formation of new hydrogen bonds in the N-domain – C-domain interface starting in the II model, and gradually increasing from the III and IV models to the ATP+RNA+C-eIF4A models. For example, we found hydrogen bond formations with the total occupancies of 0% in the I model, 45% in the II model, 540% in the III model, 740% in the IV model and 1318% in the ATP+RNA+C-eIF4A model (see Table 5). In the case of initial RNA binding followed by ATP, results of the motion correlation analysis (see blue squares of Figure S6A) for the allosteric simulation of the RNA+eIF4A model suggest that the motions of RNA-binding β3-α5/β4-β5-α6 region of N-domain, correlated with the motions of the α5-β4/β3/6/7 interface segments of the N-domain. Further, the motions of the interface segments correlated with the motions of the α3/4/5 interface helices of the C-domain. Similar correlation occurred from the RNA-binding α2/β3-α3/β4-α4 region to the interface region in C-domain, and from the C-domain interface to the N-domain interface (see green squares in Figure S6A). Such correlation suggested that the proximity between the two RNA-binding regions at N-domain and C-domain was associated with decreases in their average mass center distances from 47.1 Å in the O-eIF4A model to 31.6 Å in the RNA+eIF4A model. The effect communicated to the movement of the α5-β4/β3/6/7 interface segments of the N-domain close to the α3/4/5 interface helices of the C-domain with the decreases in their average mass center distances from 43.9 Å in the O-eIF4A model to 29.1 Å in the RNA+eIF4A model with hydrogen bond formation. For example, the hydrogen bond formation between Glu100/Lys107 of N-domain and Glu297 of C-domain was associated with occupancies of 26.20% and 14.40%, respectively (see Table S3). Such hydrogen bond formations at the N-domain – C-domain interface were absent with the model involving initial ATP binding. i.e., the ATP+eIF4A model.

In summary, the RNA allosteric network analysis demonstrates that RNA binding with eIF4A protein mediated the proximity of the two protein domains via the migration of RNA bases cooperatively assisted by ATP binding. The migration of RNA bases may facilitate the unwinding of RNA duplex in the cytoplasm.

## Conclusions

Molecular dynamics simulations and free energy calculations for a series of constructed O-eIF4A, ATP+RNA+C-eIF4A, ATP+eIF4A, RNA+eIF4A and intermediate models have been performed to explore the possible allosteric mechanisms from an open eIF4A to closed state due to ATP and RNA binding. The results for two stable open and closed eIF4A states show that the mass center distance and the relative angles of N- and C-domains in the eIF4A protein changed from 48.7 Å and 0° in the open state to 29.5 Å and 103.6° in the closed state, respectively. Based on the free energy calculations, the eIF4A protein completed the conformational transition from the open eIF4A to closed state via two allosteric mechanisms: ATP binding initially followed by RNA and vice versa. The ATP and RNA binding caused positive cooperativity by increasing the affinity of ATP and RNA of −21.70 kcal·mol^−1^ and −52.28 kcal·mol^−1^ induced by RNA and ATP binding, respectively. The two allosteric processes indicate that ATP binding to the eIF4A protein led to the relative rotation of two domains with a rotation angle of ∼60°–99°, while RNA binding caused the proximity of N- and C-domains with decreased distance of ∼12 Å via the migration of RNA bases. Initial binding by ATP with eIF4A induced the rearrangement of the RNA binding sites at the edge of N- and C-domains of eIF4A protein, which enhanced the subsequent RNA binding with the protein. The migration of RNA bases depends on the ATP binding to eIF4A protein, which favors the unwinding of the RNA duplex in the cytoplasm. It suggested that the cooperative mediation of ATP and RNA completes the conformational transition of eIF4A protein. The hydrogen bond analysis supported the conformational changes of eIF4A protein during the allosteric process. The allosteric correlation and network analysis demonstrates that the structural changes in ATP-binding region of α2-α3/β2-P-loop-α4 at N-domain communicated to allosteric changes at RNA-binding regions of β3-α5/β4-β5-α6 at N-domain and α2/β3-α3/β4-α4 at C-domain, and the N-domain – C-domain interface involved in initial ATP binding. Such network analysis of initial RNA binding suggested that the structural variation of RNA binding only communicates to the allostery of N-domain – C-domain interface. The present investigations provide useful insights into the cooperative mediation of ATP and RNA in the allosteric modulation of eIF4A protein.

## Supporting Information

Figure S1 **The sequence alignment for the yeast eIF4A and human eIF4AIII proteins.** Structure-based sequence alignment for the yeast eIF4A and human eIF4AIII. Conserved residues are colored in deep blue and nine conserved motifs (Q, I, Ia, Ib, II, III, IV, V and VI) are shown in the sub-regions squared in magenta.
(TIF)

Figure S2 **RMSD values of the intermediate models.** RMSD values of all backbone atoms with respect to the corresponding starting structures for the CMD simulations of (A) I, (B) II, (C) III, (D) IV taken from TMD simulation of the (ATP+eIF4A)+RNA→(ATP+RNA+C-eIF4A) transition and (E) I', (F) II', (G) III', (H) IV' taken from the (RNA+eIF4A)+ATP→(ATP+RNA+C-eIF4A) transition.
(TIF)

Figure S3 **The structures of the allosteric process.** The average structures are extracted from the trajectories of the O-eIF4A, RNA+eIF4A, I', II', III', IV' and ATP+RNA+C-eIF4A models involved in the allosteric process of the RNA binding followed by ATP, i.e., the RNA+eIF4A model for the RNA first binding to the O-eIF4A model; the I'–IV' models for the ATP second binding to the equilibrium structure of the RNA+eIF4A model; the ATP+RNA+C-eIF4A model for the closed state of the eIF4A protein; the average mass center distance of two domains labeled in black lines.
(TIF)

Figure S4 **Domain rotation angel between the RNA+eIF4A and ATP+RNA+C-eIF4A models.** The relative rotation angel (magenta lines) of the two domains in the eIF4A protein between the RNA+eIF4A (yellow orange) and ATP+RNA+C-eIF4A (pale cyan) models.
(TIF)

Figure S5 **The fluctuations of residues.** The fluctuations of residues in O-eIF4A (green), RNA+eIF4A (magenta) and ATP+RNA+C-eIF4A (blue) models with the labeled α helices and β strands at the ATP/RNA binding region and the N-domain – C-domain interface in the shaded regions.
(TIF)

Figure S6 **Correlation maps and the differences of correlations.** Dynamical cross-correlation maps for (A) allosteric RNA+eIF4A simulation and (B) the differences of motion correlations between the ATP+RNA+C-eIF4A and RNA+eIF4A equilibrium simulations, with specific sub-regions squared in blue for the correlations of the N-domain RNA binding region and the N-domain – C-domain interface, in green for the correlations of the C-domain RNA binding region and the N-domain – C-domain interface, in black for the correlations of the ATP binding and RNA binding bases, and in magenta for the correlations of the ATP binding region and the N-domain – C-domain interface.
(TIF)

Figure S7 **The distance and the rotation angel of two domains.** The mass center distance (black line) of two domains of the eIF4A protein in the eIF4A-eIF4G complex; the rotation angel (magenta lines) of the eIF4A protein between the O-eIF4A state (slate) and the eIF4A-eIF4G complex (cyan). The eIF4A protein shown in cartoon form and the scaffold eIF4G protein colored in cyan semi-transparent surface.
(TIF)

Table S1 **The occupancies (%) of hydrogen bonds between the RNA and the N-domain (ND) of the eIF4A protein, for the RNA+eIF4A (RNA+4A), I', II', III', IV' and ATP+RNA+C-eIF4A (C) models.**
(PDF)

Table S2 **The occupancies (%) of hydrogen bonds between the RNA and the C-domain (CD) of the eIF4A for the RNA+eIF4A (RNA+4A), I', II', III', IV' and ATP+RNA+C-eIF4A (C) models.**
(PDF)

Table S3 **The occupancies (%) of hydrogen bonds of the interface between the N-terminal domain and C-terminal domain in the RNA+eIF4A (RNA+4A), I', II', III', IV' and ATP+RNA+C-eIF4A (C) model.**
(PDF)

Text S1 **Molecular dynamics simulation protocols used in this work.**
(PDF)

Text S2 **Targeted molecular dynamics simulation protocols used in this work.**
(PDF)

Text S3 **MM-PBSA calculation for free energy.**
(PDF)

## References

[1] Duncan R, Milburn SC, Hershey JW (1987) Regulated phosphorylation and low abundance of HeLa cell initiation factor eIF-4F suggest a role in translational control. Heat shock effects on eIF-4F. J Biol Chem 262: 380–388. 3793730

[2] Spirin AS (2009) How does a scanning ribosomal particle move along the 5′-untranslated region of eukaryotic mRNA? Brownian Ratchet model. Biochemistry 48: 10688–10692.19835415

[3] Sonenberg N, Hinnebusch AG (2009) Regulation of translation initiation in eukaryotes: mechanisms and biological targets. Cell 136: 731–745. 19239892 10.1016/j.cell.2009.01.042 PMC3610329

[4] Gingras A-C, Raught B, Sonenberg N (1999) eIF4 initiation factors: effectors of mRNA recruitment to ribosomes and regulators of translation. Annu Rev Biochem 68: 913–963. 10872469 10.1146/annurev.biochem.68.1.913

[5] Pestova TV, Kolupaeva VG, Lomakin IB, Pilipenko EV, Shatsky IN, et al (2001) Molecular mechanisms of translation initiation in eukaryotes. Proc Natl Acad Sci U S A 98: 7029–7036. 11416183 10.1073/pnas.111145798 PMC34618

[6] Kapp LD, Lorsch JR (2004) The molecular mechanics of eukaryotic translation. Annu Rev Biochem 73: 657–704. 15189156 10.1146/annurev.biochem.73.030403.080419

[7] Gebauer F, Hentze MW (2004) Molecular mechanisms of translational control. Nat Rev Mol Cell Biol 5: 827–835. 15459663 10.1038/nrm1488 PMC7097087

[8] Jackson RJ, Hellen CU, Pestova TV (2010) The mechanism of eukaryotic translation initiation and principles of its regulation. Nat Rev Mol Cell Biol 11: 113–127. 20094052 10.1038/nrm2838 PMC4461372

[9] Staley JP, Guthrie C (1998) Mechanical devices of the spliceosome: motors, clocks, springs, and things. Cell 92: 315–326. 9476892 10.1016/s0092-8674(00)80925-3

[10] Venema J, Tollervey D (1995) Processing of pre-ribosomal RNA in Saccharomyces cerevisiae. Yeast 11: 1629–1650. 8720068 10.1002/yea.320111607

[11] Py B, Higgins CF, Krisch HM, Carpousis AJ (1996) A DEAD-box RNA helicase in the Escherichia coli RNA degradosome. Nature 381: 169–172. 8610017 10.1038/381169a0

[12] Pain VM (1996) Initiation of Protein Synthesis in Eukaryotic Cells. Eur J Biochem 236: 747–771. 8665893 10.1111/j.1432-1033.1996.00747.x

[13] Linder P, Gasteiger E, Bairoch A (2000) A comprehensive web resource on RNA helicases from the baker's yeast Saccharomyces cerevisiae. Yeast 16: 507–509. 10790687 10.1002/(SICI)1097-0061(200004)16:6<507::AID-YEA549>3.0.CO;2-N

[14] Svitkin YV, Pause A, Haghighat A, Pyronnet S, Witherell G, et al (2001) The requirement for eukaryotic initiation factor 4A (elF4A) in translation is in direct proportion to the degree of mRNA 5′ secondary structure. RNA 7: 382–394. 11333019 10.1017/s135583820100108x PMC1370095

[15] Jankowsky E, Fairman ME (2007) RNA helicases–one fold for many functions. Curr Opin Struct Biol 17: 316–324. 17574830 10.1016/j.sbi.2007.05.007

[16] Peck ML (2003) Adenosine 5′-O-(3-thio)triphosphate (ATP S) is a substrate for the nucleotide hydrolysis and RNA unwinding activities of eukaryotic translation initiation factor eIF4A. RNA 9: 1180–1187. 13130132 10.1261/rna.2103703 PMC1370482

[17] Andersen CB, Ballut L, Johansen JS, Chamieh H, Nielsen KH, et al (2006) Structure of the exon junction core complex with a trapped DEAD-box ATPase bound to RNA. Science 313: 1968–1972. 16931718 10.1126/science.1131981

[18] Bono F, Ebert J, Lorentzen E, Conti E (2006) The crystal structure of the exon junction complex reveals how it maintains a stable grip on mRNA. Cell 126: 713–725. 16923391 10.1016/j.cell.2006.08.006

[19] Sengoku T, Nureki O, Nakamura A, Kobayashi S, Yokoyama S (2006) Structural basis for RNA unwinding by the DEAD-box protein Drosophila Vasa. Cell 125: 287–300. 16630817 10.1016/j.cell.2006.01.054

[20] Hilbert M, Kebbel F, Gubaev A, Klostermeier D (2011) eIF4G stimulates the activity of the DEAD box protein eIF4A by a conformational guidance mechanism. Nucleic Acids Res 39: 2260–2270. 21062831 10.1093/nar/gkq1127 PMC3064780

[21] Karow AR, Klostermeier D (2010) A structural model for the DEAD box helicase YxiN in solution: localization of the RNA binding domain. J Mol Biol 402: 629–637. 20691700 10.1016/j.jmb.2010.07.049

[22] Marintchev A, Edmonds KA, Marintcheva B, Hendrickson E, Oberer M, et al (2009) Topology and regulation of the human eIF4A/4G/4H helicase complex in translation initiation. Cell 136: 447–460. 19203580 10.1016/j.cell.2009.01.014 PMC2656774

[23] Schutz P, Bumann M, Oberholzer AE, Bieniossek C, Trachsel H, et al (2008) Crystal structure of the yeast eIF4A-eIF4G complex: an RNA- helicase controlled by protein-protein interactions. Proc Natl Acad Sci U S A 105: 9564–9569. 18606994 10.1073/pnas.0800418105 PMC2474498

[24] Benz J, Trachsel H, Baumann U (1999) Crystal structure of the ATPase domain of translation initiation factor 4A from Saccharomyces cerevisiae – the prototype of the DEAD box protein family. Structure 7: 671–679. 10404596 10.1016/s0969-2126(99)80088-4

[25] Johnson ER, McKay DB (1999) Crystallographic structure of the amino terminal domain of yeast initiation factor 4A, a representative DEAD-box RNA helicase. RNA 5: 1526–1534. 10606264 10.1017/s1355838299991410 PMC1369875

[26] Caruthers JM, Johnson ER, McKay DB (2000) Crystal structure of yeast initiation factor 4A, a DEAD-box RNA helicase. Proc Natl Acad Sci U S A 97: 13080–13085. 11087862 10.1073/pnas.97.24.13080 PMC27181

[27] Nielsen KH, Chamieh H, Andersen CB, Fredslund F, Hamborg K, et al (2009) Mechanism of ATP turnover inhibition in the EJC. RNA 15: 67–75. 19033377 10.1261/rna.1283109 PMC2612766

[28] Collins R, Karlberg T, Lehtio L, Schutz P, van den Berg S, et al (2009) The DEXD/H-box RNA helicase DDX19 is regulated by an {alpha}-helical switch. J Biol Chem 284: 10296–10300. 19244245 10.1074/jbc.C900018200 PMC2667716

[29] von Moeller H, Basquin C, Conti E (2009) The mRNA export protein DBP5 binds RNA and the cytoplasmic nucleoporin NUP214 in a mutually exclusive manner. Nat Struct Mol Biol 16: 247–254. 19219046 10.1038/nsmb.1561

[30] Lorsch JR, Herschlag D (1998) The DEAD Box Protein eIF4A. 1. A Minimal Kinetic and Thermodynamic Framework Reveals Coupled Binding of RNA and Nucleotide. Biochemistry 37: 2180–2193. 9485364 10.1021/bi972430g

[31] Lorsch JR, Herschlag D (1998) The DEAD Box Protein eIF4A. 2. A Cycle of Nucleotide and RNA-Dependent Conformational Changes. Biochemistry 37: 2194–2206. 9485365 10.1021/bi9724319

[32] Theissen B, Karow AR, Kohler J, Gubaev A, Klostermeier D (2008) Cooperative binding of ATP and RNA induces a closed conformation in a DEAD box RNA helicase. Proc Natl Acad Sci U S A 105: 548–553. 18184816 10.1073/pnas.0705488105 PMC2206573

[33] Andreou AZ, Klostermeier D (2012) Conformational changes of DEAD-box helicases monitored by single molecule fluorescence resonance energy transfer. Methods Enzymol 511: 75–109. 22713316 10.1016/B978-0-12-396546-2.00004-8

[34] Hilbert M, Karow AR, Klostermeier D (2009) The mechanism of ATP-dependent RNA unwinding by DEAD box proteins. Biol Chem 390: 1237–1250. 19747077 10.1515/BC.2009.135

[35] Andreou AZ, Klostermeier D (2013) The DEAD-box helicase eIF4A: paradigm or the odd one out? RNA Biol 10: 19–32. 22995829 10.4161/rna.21966 PMC3590233

[36] Aregger R, Klostermeier D (2009) The DEAD box helicase YxiN maintains a closed conformation during ATP hydrolysis. Biochemistry 48: 10679–10681. 19839642 10.1021/bi901278p

[37] Liu F, Putnam A, Jankowsky E (2008) ATP hydrolysis is required for DEAD-box protein recycling but not for duplex unwinding. Proc Natl Acad Sci U S A 105: 20209–20214. 19088201 10.1073/pnas.0811115106 PMC2629341

[38] Klostermeier D (2011) Single-molecule FRET reveals nucleotide-driven conformational changes in molecular machines and their link to RNA unwinding and DNA supercoiling. Biochem Soc Trans 39: 611–616. 21428949 10.1042/BST0390611

[39] Li Q, Imataka H, Morino S, Rogers GW, Richter-Cook NJ, et al (1999) Eukaryotic translation initiation factor 4AIII (eIF4AIII) is functionally distinct from eIF4AI and eIF4AII. Mol Cell Biol 19: 7336–7346. 10523622 10.1128/mcb.19.11.7336 PMC84727

[40] Jaya Herlambang S (2012) Build mutant and build homology protein structure predictions for indonesian avian influenza neuraminidase. J Biol Chem 03: 183–190.

[41] Case DA, Darden TA, Cheatham TE III, Simmerling CL, Wang JM, et al.. (2006) University of California, San Francisco.

[42] Duan Y, Wu C, Chowdhury S, Lee MC, Xiong G, et al (2003) A point-charge force field for molecular mechanics simulations of proteins based on condensed-phase quantum mechanical calculations. J Comput Chem 24: 1999–2012. 14531054 10.1002/jcc.10349

[43] Lee MC, Duan Y (2004) Distinguish protein decoys by using a scoring function based on a new AMBER force field, short mole nnnnnnn cular dynamics simulations, and the generalized born solvent model. Proteins 55: 620–634. 15103626 10.1002/prot.10470

[44] Perez A, Marchan I, Svozil D, Sponer J, Cheatham TE III, et al (2007) Refinement of the AMBER force field for nucleic acids: improving the description of alpha/gamma conformers. Biophys J 92: 3817–3829. 17351000 10.1529/biophysj.106.097782 PMC1868997

[45] Wang J, Wolf RM, Caldwell JW, Kollamn PA, Case DA (2004) Development and testing of a general Amber force field. J Comput Chem 25: 1157–1174. 15116359 10.1002/jcc.20035

[46] Cheatham TE, Srinivasan J, Case DA, Kollman PA (1998) Molecular dynamics and continuum solvent studies of the stability of polyG-polyC and polyA-polyT DNA duplexes in solution. J Biomol Stuct Dyn 16: 265–280. 10.1080/07391102.1998.10508245 9833666

[47] Kollman PA, Massova I, Reyes C, Kuhn B, Huo S, et al (2000) Calculating structures and free energies of complex molecules: combining molecular mechanics and continuum models. Acc Chem Res 33: 889–897. 11123888 10.1021/ar000033j

[48] Jayaram B, Sprous D, Young MA, Beveridge DL (1998) Free energy analysis of the conformational preferences of A and B forms of DNA in solution. J Am Chem Soc 120: 10629–10633 49. 47.

[49] Srinivasan JCTI, Cieplak P, Kollman PA, Case DA (1998) Continuum solvent studies of the stability of DNA, RNA, and phosphoramidate DNA helices. J Am Chem Soc 120: 9401–9409.

[50] McQuarrie DA (1976) Statistical mechanics. Harper & Row, NY.

[51] Kottalam J, Case DA (1990) Langevin modes of macromolecules: Applications to crambin and DNA hexamers. Biopolymers 29: 1409–1421. 2361153 10.1002/bip.360291008

[52] Jensen F (1999) Introduction to computational chemistry. Wiley, Chichester.

[53] Hayward S, Berendsen HJC (1998) Systematic analysis of domain motions in protein from conformational change: new results on citrate synthase and T4 lysozyme. Proteins: Struct Funct Genet 30: 144–154. 9489922

[54] Yap KL, Ames JB, Swindells MB, Ikura M (1999) Diversity of Conformational States and Changes Within the EF-Hand Protein Superfamily. Proteins 37: 499–507. 10591109 10.1002/(sici)1097-0134(19991115)37:3<499::aid-prot17>3.0.co;2-y

[55] Yap KL, Ames JB, Swindells MB, Ikura M (2001) Vector Geometry Mapping: A Method to Characterize the Conformation of Helix-Loop-Helix Calcium Binding Proteins. Methods Mol Biol 173: 1–9. 10.1385/1-59259-184-1:317 11859772

[56] Sadiq SK, Fabritiis GD (2010) Explicit solvent dynamics and energetics of HIV-1 protease flap opening and closing. Proteins: Struct Funct Genet 78: 2873–2885. 20715057 10.1002/prot.22806

[57] Brocklehurst SM, Perham RN (1993) Prediction of the three-dimensional structures of the biotinylated domain from yeast pyruvate carboxylase and of the lipoylated H-protein from the pea leaf glycine cleavage system: A new automated method for the prediction of protein tertiary structure. Protein Sci 2: 626–639. 8518734 10.1002/pro.5560020413 PMC2142356

[58] Chuprina V, Rullmann J, Lamerichs R, Van Boom J, Boelens R, et al (1993) Structure of the complex of lac repressor headpiece and an 11 base-pair half-operator determined by nuclear magnetic resonance spectroscopy and restrained molecular dynamics. J Mol Biol 234: 446. 8230225 10.1006/jmbi.1993.1598

[59] Hogbom M, Collins R, van den Berg S, Jenvert RM, Karlberg T, et al (2007) Crystal structure of conserved domains 1 and 2 of the human DEAD-box helicase DDX3X in complex with the mononucleotide AMP. J Mol Biol 372: 150–159. 17631897 10.1016/j.jmb.2007.06.050

