# Supplementary figures and images for: Molecular Dynamics Simulation of the Allosteric Regulation of eIF4A Protein from the Open to Closed State, Induced by ATP and RNA Substrates
Source: PLoS One. 2014 Jan 23;9(1):e86104. doi: 10.1371/journal.pone.0086104 (PMC3900488; doi:10.1371/journal.pone.0086104)

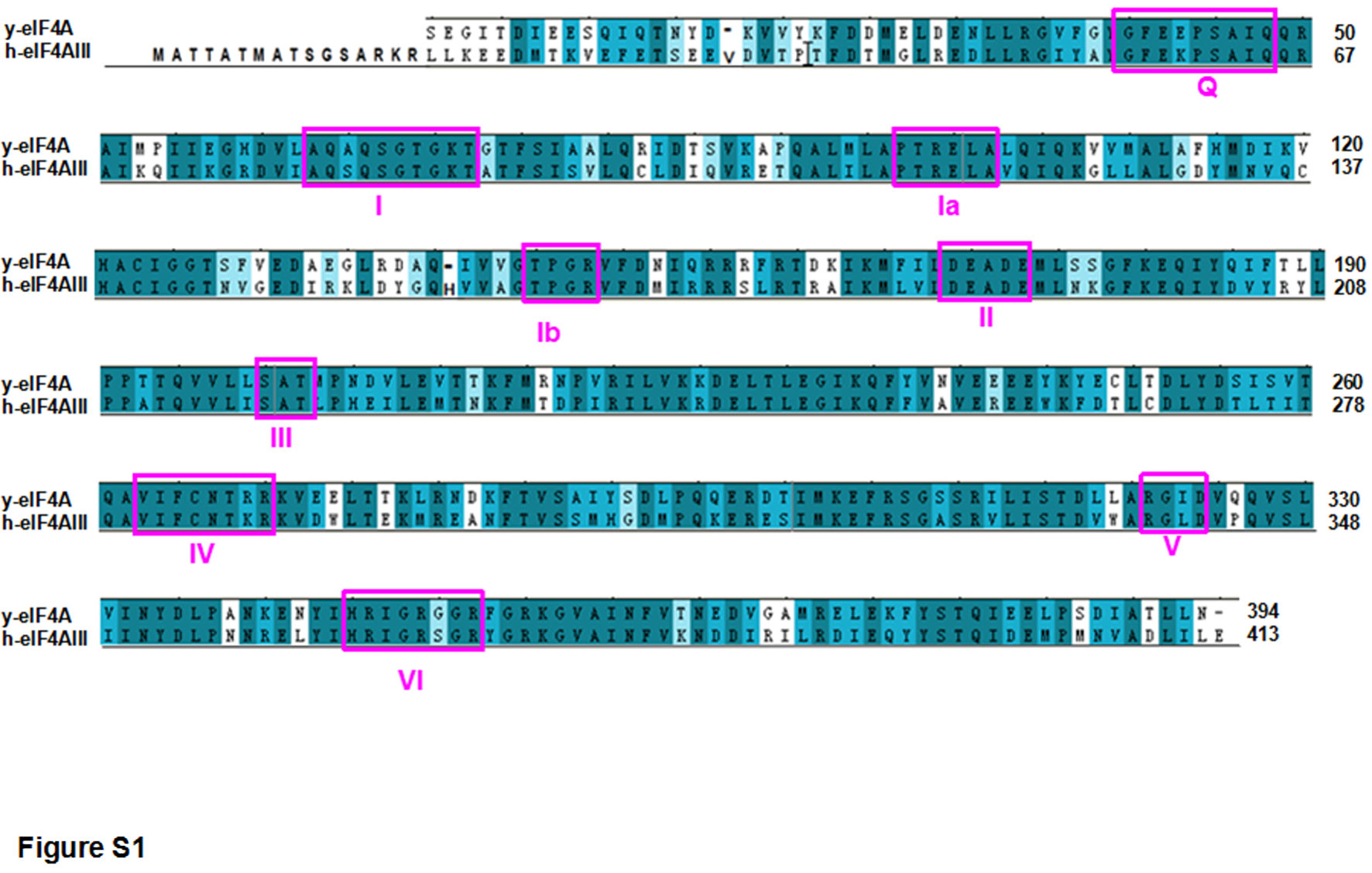

Supplement: Figure S1 — The sequence alignment for the yeast eIF4A and human eIF4AIII proteins. Structure-based sequence alignment for the yeast eIF4A and human eIF4AIII. Conserved residues are colored in deep blue and nine conserved motifs (Q, I, Ia, Ib, II, III, IV, V and VI) are shown in the sub-regions squared in magenta. (TIF) [file pone.0086104.s001.tif]

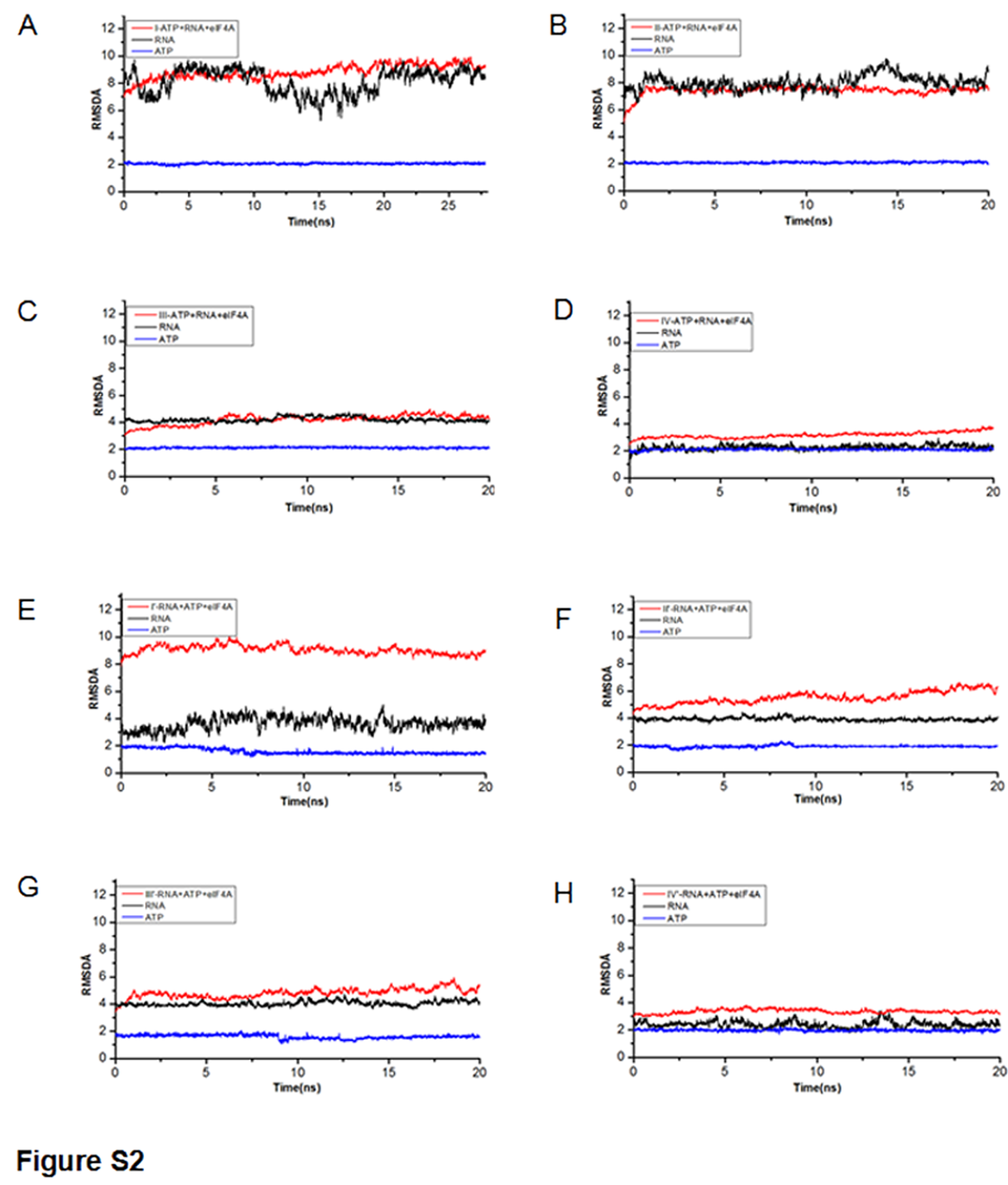

Supplement: Figure S2 — RMSD values of the intermediate models. RMSD values of all backbone atoms with respect to the corresponding starting structures for the CMD simulations of (A) I, (B) II, (C) III, (D) IV taken from TMD simulation of the (ATP+eIF4A)+RNA→(ATP+RNA+C-eIF4A) transition and (E) I', (F) II', (G) III', (H) IV' taken from the (RNA+eIF4A)+ATP→(ATP+RNA+C-eIF4A) transition. (TIF) [file pone.0086104.s002.tif]

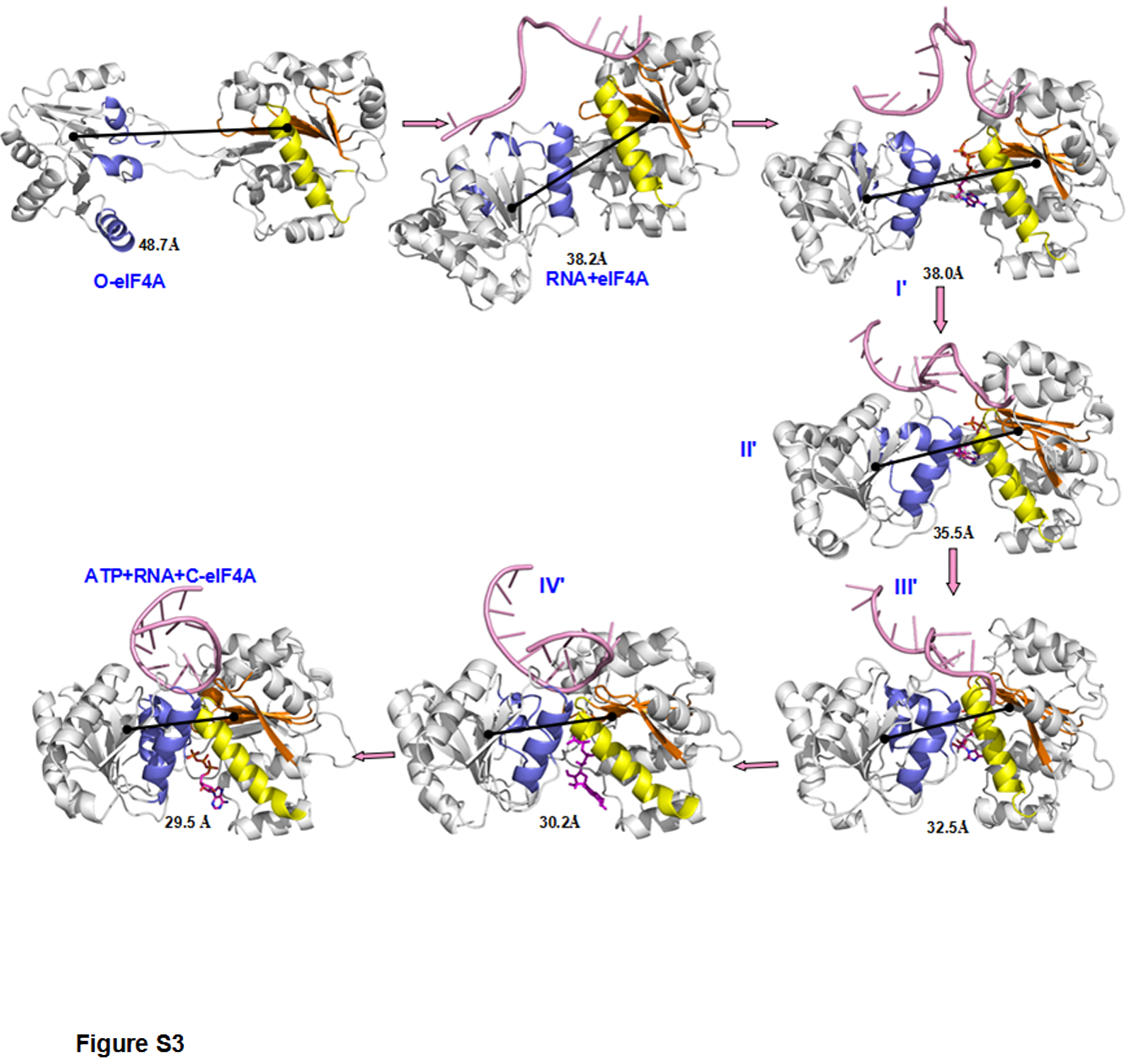

Supplement: Figure S3 — The structures of the allosteric process. The average structures are extracted from the trajectories of the O-eIF4A, RNA+eIF4A, I', II', III', IV' and ATP+RNA+C-eIF4A models involved in the allosteric process of the RNA binding followed by ATP, i.e., the RNA+eIF4A model for the RNA first binding to the O-eIF4A model; the I'–IV' models for the ATP second binding to the equilibrium structure of the RNA+eIF4A model; the ATP+RNA+C-eIF4A model for the closed state of the eIF4A protein; the average mass center distance of two domains labeled in black lines. (TIF) [file pone.0086104.s003.tif]

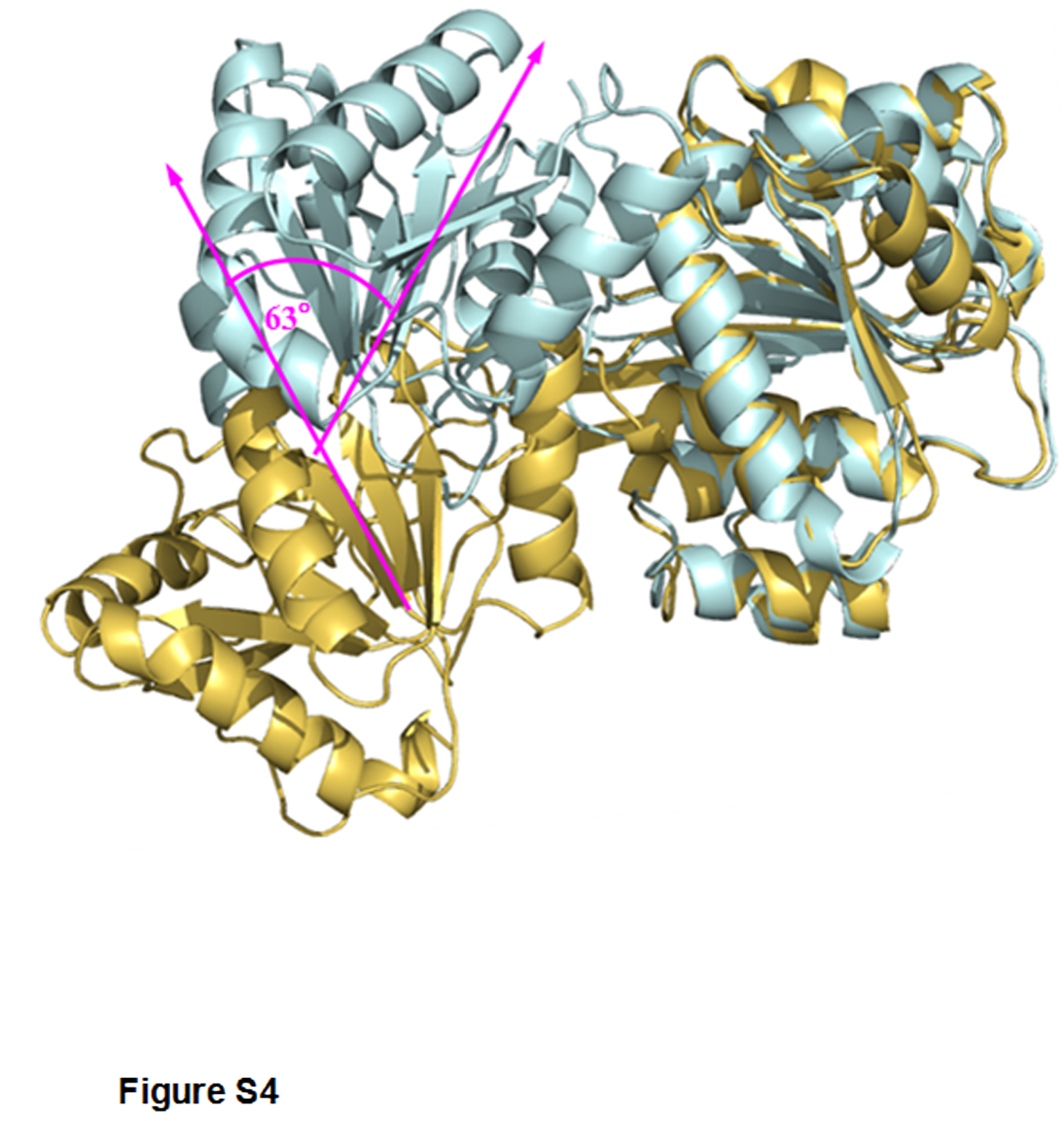

Supplement: Figure S4 — Domain rotation angel between the RNA+eIF4A and ATP+RNA+C-eIF4A models. The relative rotation angel (magenta lines) of the two domains in the eIF4A protein between the RNA+eIF4A (yellow orange) and ATP+RNA+C-eIF4A (pale cyan) models. (TIF) [file pone.0086104.s004.tif]

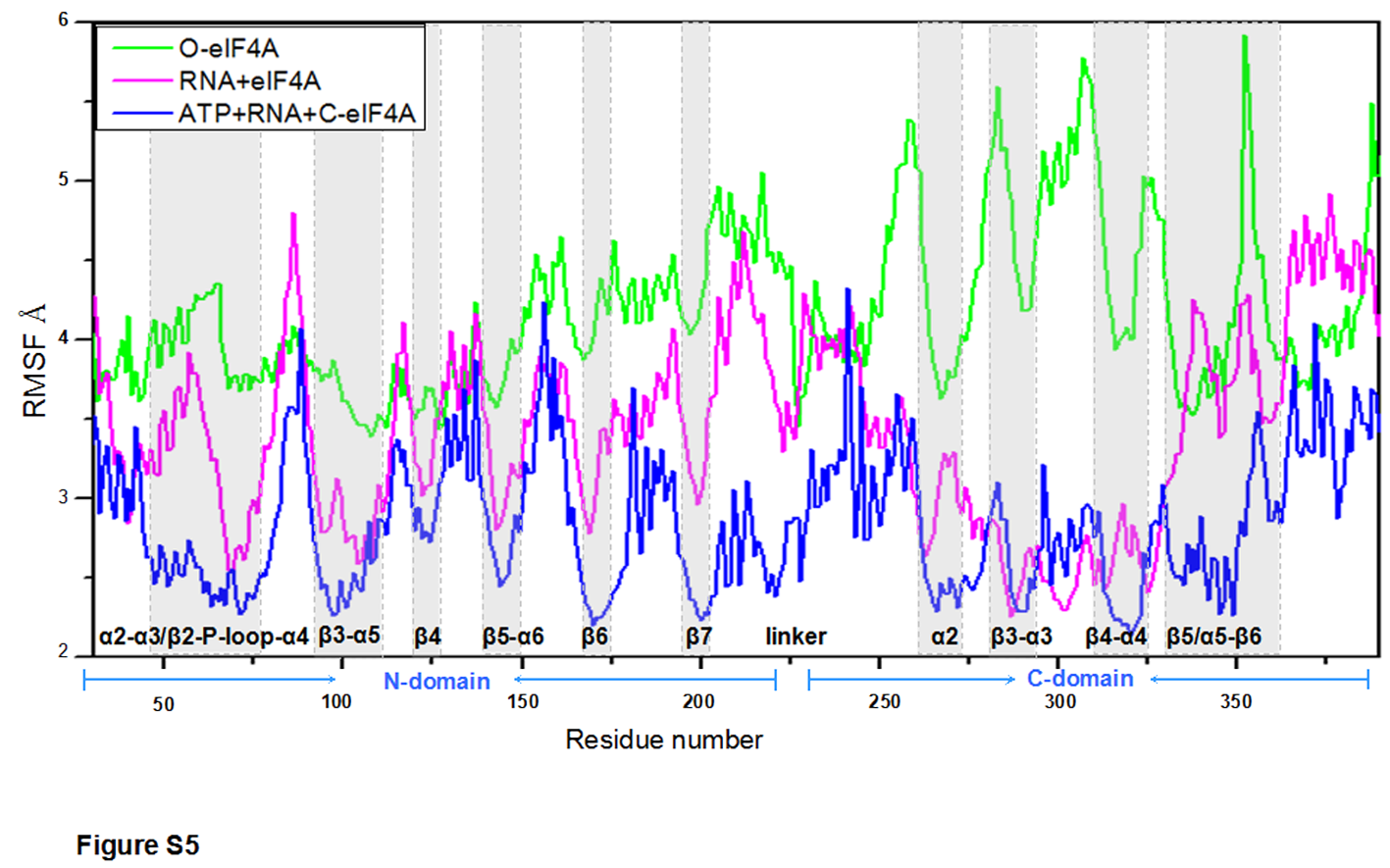

Supplement: Figure S5 — The fluctuations of residues. The fluctuations of residues in O-eIF4A (green), RNA+eIF4A (magenta) and ATP+RNA+C-eIF4A (blue) models with the labeled α helices and β strands at the ATP/RNA binding region and the N-domain – C-domain interface in the shaded regions. (TIF) [file pone.0086104.s005.tif]

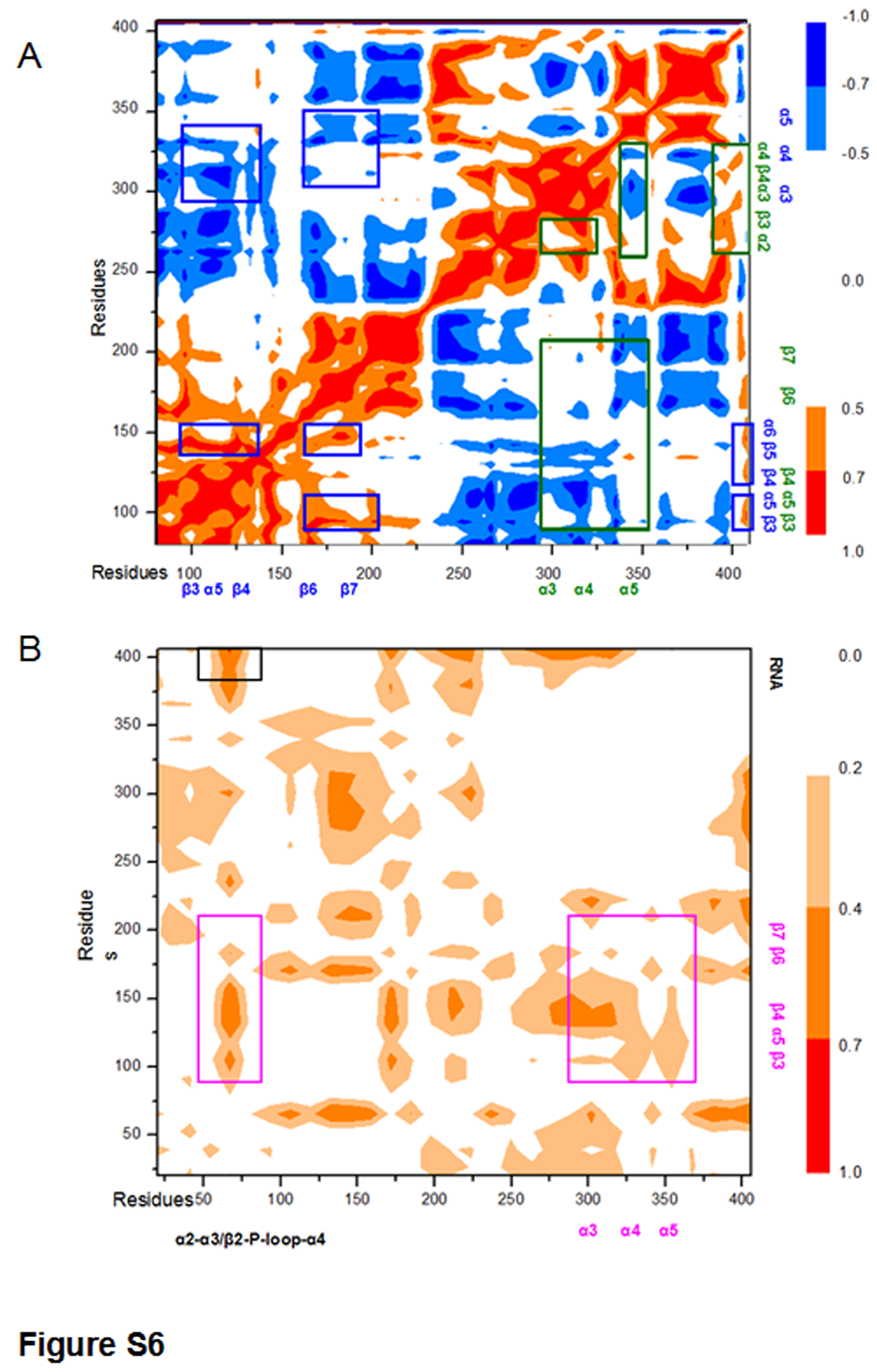

Supplement: Figure S6 — Correlation maps and the differences of correlations. Dynamical cross-correlation maps for (A) allosteric RNA+eIF4A simulation and (B) the differences of motion correlations between the ATP+RNA+C-eIF4A and RNA+eIF4A equilibrium simulations, with specific sub-regions squared in blue for the correlations of the N-domain RNA binding region and the N-domain – C-domain interface, in green for the correlations of the C-domain RNA binding region and the N-domain – C-domain interface, in black for the correlations of the ATP binding and RNA binding bases, and in magenta for the correlations of the ATP binding region and the N-domain – C-domain interface. (TIF) [file pone.0086104.s006.tif]

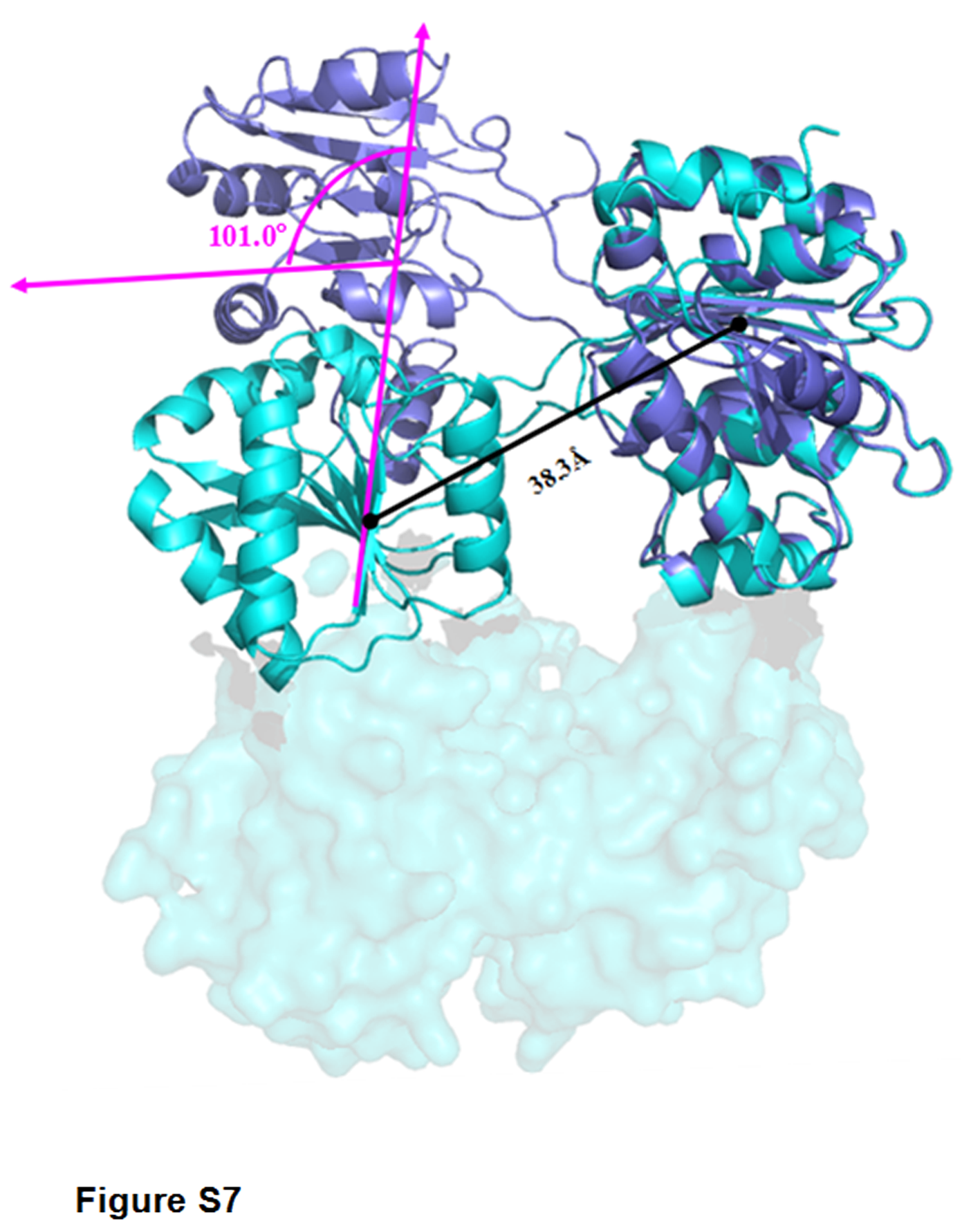

Supplement: Figure S7 — The distance and the rotation angel of two domains. The mass center distance (black line) of two domains of the eIF4A protein in the eIF4A-eIF4G complex; the rotation angel (magenta lines) of the eIF4A protein between the O-eIF4A state (slate) and the eIF4A-eIF4G complex (cyan). The eIF4A protein shown in cartoon form and the scaffold eIF4G protein colored in cyan semi-transparent surface. (TIF) [file pone.0086104.s007.tif]
