# Supplementary material for: Molecular Dynamics Simulation of the Allosteric Regulation of eIF4A Protein from the Open to Closed State, Induced by ATP and RNA Substrates
Source: PLoS One. 2014 Jan 23;9(1):e86104. doi: 10.1371/journal.pone.0086104 (PMC3900488; doi:10.1371/journal.pone.0086104)
Supplement: Table S1 — The occupancies (%) of hydrogen bonds between the RNA and the N-domain (ND) of the eIF4A protein, for the RNA+eIF4A (RNA+4A), I', II', III', IV' and ATP+RNA+C-eIF4A (C) models. (PDF) [file pone.0086104.s008.pdf]

Table S1. The occupancies (%) of hydrogen bonds between the RNA and the N-domain (ND) of the eIF4A protein, for the RNA+eIF4A (RNA+4A), I’, II’, III’, IV’ and ATP+RNA+C-eIF4A (C) models.

ND	Hydrogen bond	RNA+4A	I’	II’	Hydrogen bond	III’	IV’	C
β3-α5	(U404)O2'···O-H(Pro97)	15.10			(U400)O2'···O-H(Pro97)		78.90	80.12
	(U405)O1P-H···OG1(Thr98)	29.65			(U401)O1P-H···OG1(Thr98)	9.22		96.54
	(U406)O2P-H···NH2(Arg99)	99.46	87.58	64.16	(U402)O2P-H···NH2(Arg99)		99.4	99.18
	(U406)O2P-H···NE(Arg99)	83.14	99.24	97.06	(U402) O2P-H···NE(Arg99)			98.40
	(U406)O1P-H···NE(Arg99)	80.80	14.02		(U402) O1P-H···NE(Arg99)		98.66	21.70
	(U406) P-H···NE(Arg99)	17.94	11.06	8.04	(U401) O1P-H···N(Arg99)		89.58	99.78
	(U406)O5’-H···NH2(Arg99)		69.26	5.44	(U403)O4-H···NH2(Arg99)	75.38		
	(U405)O3’-H···NH2(Arg99)			70.90	(U403)O4-H···NH1(Arg99)	46.24		
	(U405)O3’-H···NE(Arg99)			46.44	(U401)O2-H···NH2(Arg99)	24.24		
					(U401)N3···H-OE2(Glu100)	41.62		
β4-β5-α6	(U406)O1P-H···N(Gly125)	99.80	98.94	99.22	(U402)O1P-H···N(Gly125)		98.33	82.06
	(U405)O3'-H···OG1(Thr145)	45.81	26.96		(U403)O1P-H···N(Gly126)			99.82
	(U406)O1P-H···OG1(Thr145)	99.62	99.84	98.86	(U403)O2P-H···N(Gly126)		98.98	
	(U406) P-H···OG1(Thr145)	77.12	51.88	22.04	(U406)O2’-H···N(Ser128)	23.60		
	(U405)O2'···H-OG1(Thr145)	54.19	85.14		(U406)O2’-H···OE2(Glu131)	60.62		
	(U405) O2'-H···N(Gly147)	85.44	75.94		(U401)O3'-H···OG1(Thr145)		65.43	22.40
	(U406) N3···H-O(Gly147)		58.10		(U402)O1P-H···OG1(Thr145)		99.96	100.0
	(U405) O2'-H···N(Arg148)	43.79	59.64		(U402) P-H···OG1(Thr145)		18.16	34.16
	(U406)O3'-H···NH1(Arg148)	84.76		33.78	(U401) O2'-H···N(Gly147)		81.42	40.46
	(U406)O4'-H···NH1(Arg148)		19.44		(U402)O2'-H···NE(Arg148)			69.26
	(U404)N3-H···O(Gly178)		92.60		(U402)O1P-H···N(Arg148)			64.10
	(U405) O2-H···NE2(Gln182)	21.16	69.96		(U402) O3'-H···NE(Arg148)		24.98	43.26
					(U403)O1P-H···NH2(Arg148)			97.66
					(U403)O2P-H···NH2(Arg148)		45.83	
					(U402)O2-H···NH1(Arg148)		71.39	
					(U402)O2'-H···OD1(Arg151)		75.94	33.28
					(U399)O4-H···N(Gly178)		48.43	
