# Supplementary material for: Molecular Dynamics Simulation of the Allosteric Regulation of eIF4A Protein from the Open to Closed State, Induced by ATP and RNA Substrates
Source: PLoS One. 2014 Jan 23;9(1):e86104. doi: 10.1371/journal.pone.0086104 (PMC3900488; doi:10.1371/journal.pone.0086104)
Supplement: Table S2 — The occupancies (%) of hydrogen bonds between the RNA and the C-domain (CD) of the eIF4A for the RNA+eIF4A (RNA+4A), I', II', III', IV' and ATP+RNA+C-eIF4A (C) models. (PDF) [file pone.0086104.s009.pdf]

Table S2. The occupancies (%) of hydrogen bonds between the RNA and the C-domain (CD) of the eIF4A for the RNA+eIF4A (RNA+4A), I’, II’, III’, IV’ and ATP+RNA+C-eIF4A (C) models.

CD	Hydrogen bond	RNA+4A	I’	II’	Hydrogen bond	III’	IV’	C
α2	(U395)O2'···H-O(Pro267)		41.14		(U398)O2'···H-O(Pro267)		79.96	66.50
	(U396)O4-H···ND2 (Asn267)	22.20			(U399)O1P-H···N(Arg269)	33.08	99.28	94.72
	(U398)O1P-H··OG1(Thr268)			97.08	(U399)O2P-H···NH2(Arg269)	79.36	90.20	50.30
	(U395) O2'-H···N(Arg269)	66.59			(U399)O1P-H···NE(Arg269)	62.82	83.82	36.72
	(U395) O3'-H···NH1(Arg269)	14.36			(U399) P-H···NE(Arg269)	47.14	32.55	9.70
	(U401) O3'-H···NH1(Arg269)			22.00	(U399)O5'-H···NE(Arg269)		23.66	9.66
	(U396)O1P-H···N(Arg269)	85.10	68.86		(U399)O2P-H···NE(Arg269)		32.37	17.94
	(U396) P-H···NH1(Arg269)	20.08			(U400) O3'-H···NH2(Arg269)	18.16		
	(U401)O1P-H···NH1(Arg269)			40.10	(U398) O1P-H···N(Arg270)	47.42		
	(U402) O1P-H···NH2(Arg269)			76.40	(U398) O2P-H···NE(Arg270)	25.74		
	(U401) O1P-H···NE(Arg269)			69.28	(U397)O1P-H···NH2(Arg270)	19.22		
	(U395) O1P-H···N(Arg270)	76.11						
	(U398) O1P-H···NE(Arg270)			94.94				
	(U398) O1P-H···NH2(Arg270)			60.12				
β3-α3	(U397) O1P-H···OG(Ser291)	62.91	86.50		(U400)O1P-H···OG(Ser291)		16.26	21.88
	(U400) O2'-H···OG(Ser291)			16.14	(U400) P-H···OG(Ser291)		53.83	61.46
	(U404) O2'···H-O(Ser291)			40.04	(U400)O2P-H···OG(Ser291)		99.18	99.34
	(U396) O3'-H···OG(Ser291)	72.65			(U400)O1P-H···N(Ser291)		99.64	100.0
	(U404) O2'-H···NH2(Arg298)			36.44	(U400) O2-H···OG(Ser291)	37.96		
					(U401)O1P-H···NH2(Arg298)			99.76
					(U401)O2P-H···NH1(Arg298)		9.30	99.02
					(U401)O2P-H···NH2(Arg298)			52.42
					(U401) O2-H···NH2(Arg298)	65.62		
β4-α4	(U396) O2-H···N(Leu318)	94.30			(U399)O3'-H···OG1(Thr316)		72.05	58.88
					(U400) P-H···OG1(Thr316)			41.86
					(U400)O1P-H···OG1(Thr316)			95.24
					(U396) O2’-H···N(Leu318)		50.57	
