# Supplementary material for: Molecular Dynamics Simulation of the Allosteric Regulation of eIF4A Protein from the Open to Closed State, Induced by ATP and RNA Substrates
Source: PLoS One. 2014 Jan 23;9(1):e86104. doi: 10.1371/journal.pone.0086104 (PMC3900488; doi:10.1371/journal.pone.0086104)
Supplement: Table S3 — The occupancies (%) of hydrogen bonds of the interface between the N-terminal domain and C-terminal domain in the RNA+eIF4A (RNA+4A), I', II', III', IV' and ATP+RNA+C-eIF4A (C) model. (PDF) [file pone.0086104.s010.pdf]

Table S3. The occupancies (%) of hydrogen bonds of the interface between the N-terminal domain and C-terminal domain in the RNA+eIF4A (RNA+4A), I', II', III', IV' and ATP+RNA+C-eIF4A (C) model.

Hydrogen bond	RNA+4A	I'	II'	III'	IV'	C
(99Arg) N···H-O(295Gln)	0	0	56.82	28.98	85.20	99.82
(100Glu) O-H···N(295Gln)	0	0	13.38	20.78	0	0
(100Glu) O-H···N(296Gln)	26.20	20.66	0	0	0	0
(100Glu) O-H···N(298Arg)	0	0	0	104.14	0	207.16
(100Glu) O-H···N(Thr306)	0	0	0	0	274.44	0
(104Gln) O-H···N(296Gln)	13.70	30.16	0	0	0	0
(104Gln) O-H···N(298Arg)	0	0	45.22	0	0	0
(104Gln) N-H···N(324Asp)	0	0	0	0	2.68	15.28
(104Gln) O-H···N(324Asp)	0	0	0	14.80	0	115.14
(104Gln) N···H-O(324Asp)	0	0	11.92	0	0	0
(104Gln) N-H···O(322Gly)	0	0	0	10.46	97.80	45.58
(107Lys)N···H-O(297Glu)	14.40	0	0	0	0	0
(170Glu) O-H···N(321Arg)	0	0	0	0	0.02	99.50
(170Glu) O-H···N(345Hie)	0	0	0	0	0.02	135.38
(172Asp) O-H···N(321Arg)	0	0	0	0	0	288.08
(173Glu) O-H···N(321Arg)	0	0	0	0	128.82	183.18
(200Ser) O-H···N(321Arg)	0	0	0	0	0	89.06
(202Thr)O-H···N(321Arg)	0	0	0	0	0	40.2
