# Supplementary material for: Molecular Dynamics Simulation of the Allosteric Regulation of eIF4A Protein from the Open to Closed State, Induced by ATP and RNA Substrates
Source: PLoS One. 2014 Jan 23;9(1):e86104. doi: 10.1371/journal.pone.0086104 (PMC3900488; doi:10.1371/journal.pone.0086104)
Supplement: Text S2 — Targeted molecular dynamics simulation protocols used in this work. (PDF) [file pone.0086104.s012.pdf]

Text S2

Targeted molecular dynamics simulation

Targeted MD simulation [1,2] is a method to observe large-scale conformational transition between two known end-point conformations of a molecule. A restraint energy term was added to the energy function proportional to the square of the difference which may be characterized as the mass-weighted root-mean-square deviation (RMSD) of the current structure to the target structure in terms of atomic positions. The functional form of the restraint energy can be written as

$$E_{TMD} = \frac{1}{2} kN \left[RMSD(t) - RMSD_0(\mathrm{t}) \right]^2$$

where, k is the harmonic force constant per atom, N is the number of the restrained atoms, $RMSD(t)$ is the root-mean-square deviation of the simulated structure at time t relative to the target structure, and $RMSD_0(t)$ is the prescribed target RMSD value at time t that decreases to zero linearly with time to drive the system from an initial structure to the target structure. In the present study we use $k = 0.5$ kcal/(mol ·Å^2) as the harmonic force constant applied to all the backbone atoms of ATP, RNA and the eIF4A protein to bias the trajectories toward the target structure.

References

1. Schlitter J, Engels M, Krüger P, Jacoby E, Wollmer A (1993) Targeted molecular dynamics simulation of conformational change-application to the T↔ R transition in insulin. Mol Simul 10: 291-308.
2. Schlitter J, Engels M, Krüger P (1994) Targeted molecular dynamics: a new approach for searching pathways of conformational transitions. J Mol Graph 12: 84-89.
