# Supplementary material for: Molecular Dynamics Simulation of the Allosteric Regulation of eIF4A Protein from the Open to Closed State, Induced by ATP and RNA Substrates
Source: PLoS One. 2014 Jan 23;9(1):e86104. doi: 10.1371/journal.pone.0086104 (PMC3900488; doi:10.1371/journal.pone.0086104)
Supplement: Text S3 — MM-PBSA calculation for free energy. (PDF) [file pone.0086104.s013.pdf]

Text S3

MM-PBSA calculation for free energy

Energetic post-process was performed for each MM-PBSA calculation by using the MM-PBSA module of AMBER9 program through molecular mechanics and a continuum solvent model [1]. In MM-PBSA calculation, $G_{np/solv}$ is non-polar solvation free energy, which was calculated by using a solvent accessible surface area (SASA) as follows:

$$G_{np/solv} = rSASA + b$$

The SASA is the solvent-accessible surface area, and is estimated using Sanner's algorithm implemented in the Molsurf program in AMBER9 [2] with a probe radius of 1.4 Å. The surface tension proportionality constant (r) and the free energy of non-polar solvation for a point solute (b) are set to 0.00542 kcal mol^{-1} $Å^{-2}$ and 0.92 kcal mol^{-1}, respectively.

For each model, the last 20ns trajectory of the production dynamics stage was used for binding free energy calculations of MM-PBSA, namely, the 5000 snapshots of each model at a 4-ps interval for computation of enthalpy and 20 snapshots at 1000-ps intervals for computation of entropy.

References

1. Case DA, Darden TA, Cheatham ITE, Simmerling CL, Wang JM, et al (2006) University of California, San Francisco.
2. Connolly ML (1983) Analytical molecular surface calculation. J Appl Cryst 16: 548-558.
